# Quadrics and Scherk towers

**DOI:** 10.1007/s00605-017-1075-5

**Published:** 2017-07-03

**Authors:** S. Fujimori, U. Hertrich-Jeromin, M. Kokubu, M. Umehara, K. Yamada

**Affiliations:** 1 Department of Mathematics, Okayama University, Okayama, 700-8530 Japan; 2 Vienna University of Technology, Wiedner Hauptstraße 8–10/104, 1040 Vienna, Austria; 3 Department of Mathematics, Tokyo Denki University, Tokyo, 120-8551 Japan; 4 Department of Mathematical and Computing Sciences, Tokyo Institute of Technology, Tokyo, 152-8552 Japan; 5 Department of Mathematics, Tokyo Institute of Technology, Tokyo, 152-8551 Japan

**Keywords:** Isothermic surface, Christoffel transformation, Minimal surface, Maximal surface, Saddle tower, Scherk surface, Karcher saddle tower, Central quadric, Ellipsoid, Hyperboloid, Timelike surface, Causal type, 53C42, 53A10, 53A30, 37K25, 37K35

## Abstract

We investigate the relation between quadrics and their Christoffel duals on the one hand, and certain zero mean curvature surfaces and their Gauss maps on the other hand. To study the relation between timelike minimal surfaces and the Christoffel duals of 1-sheeted hyperboloids we introduce para-holomorphic elliptic functions. The curves of type change for real isothermic surfaces of mixed causal type turn out to be aligned with the real curvature line net.

## Introduction

Though quadrics belong to the most thoroughly investigated surfaces there are still mysterious aspects in their geometry. In particular, quadrics can reasonably be studied in a variety of ambient geometries—Möbius or conformal geometry *not* being one of these geometries since Möbius transformations generally do not map a quadric to another quadric. Nevertheless, quadrics belong to the (Möbius geometric) class of isothermic surfaces, and display rather interesting and surprising features in this context, which are far from being understood.

This paper aims to shed some light on some of these, seemingly incongruous, features.

Minimal surfaces in Euclidean space admit, away from umbilics, conformal curvature line parameters: this characterizes minimal surfaces as *isothermic surfaces*, that is, surfaces that are “capable of division into infinitesimal squares by means of their curves of curvature”, cf. [1]. Motivated by the existence of the 1-parameter family of associated minimal surfaces for a given minimal surface, that are isometric and feature parallel tangent planes, Christoffel classified those surfaces that admit a non-trivial partner surface that is conformally related to the first by parallel tangent planes, cf. [2]: apart from associated minimal surfaces these are precisely the isothermic surfaces, which admit a partner surface that is generically unique up to scaling so that the relation is orientation reversing: we will refer to this partner surface of an isothermic surface as its *Christoffel dual*. Given an isothermic surface $z\mapsto x(z)$ parametrized by conformal curvature line parameters $z=u+iv$, that is,

$$\begin{aligned} (x_z,x_z) = 0 \;{\mathrm{and}}\; \det (x_{uv},x_u,x_v) = 0, \end{aligned}$$

its Christoffel dual $z\mapsto x^*(z)$ may be obtained by integrating *Christoffel’s equations*

1.1 $$\begin{aligned} x^*_z = {1\over (x_z,x_{\bar{z}})}\,x_{\bar{z}}, \end{aligned}$$

cf. (3.2), see [2, IV] or [8, §5.2.1]. Any minimal surface yields an example, with its Gauss map providing the Christoffel dual—the reconstruction of the minimal surface from its Gauss image is essentially the Enneper–Weierstrass representation, cf [8, §5.3.12].

It is well known that quadrics are isothermic surfaces, see [1], and their Christoffel duals were determined in [15], in terms of the usual elliptic coordinates. A formulation using Jacobi elliptic functions, cf. [8, §5.2.21], allows to study the global behaviour of the ellipsoid as an isothermic surface, in particular, of its Christoffel dual and a common polarization—a holomorphic quadratic differential whose existence yields another characterization of isothermic surfaces, see [8, §5.2.12] and [17, Sect. 4].

The present paper was motivated by the observation that the Christoffel dual of a tri-axial ellipsoid is the affine image of a Scherk tower, cf. [8, §5.2.21 Footnote 21], see [16] and [14, §83(41)]. As the notions of isothermic surface and Christoffel duality are Möbius geometric and Euclidean notions, respectively, this feature of the ellipsoid and its Christoffel dual appear to be a highly unlikely coincidence: the principal aim of this paper is to shed further light on this coincidence and to obtain a better understanding of the reasons behind it.

As a key result we derive Lemma 3.2, which presents an approach to understand the phenomenon, and which allows us to easily derive similar results for 2- and 1-sheeted hyperboloids in Minkowski space, cf. [13] and [12, Prop 3.1]. As the Enneper–Weierstrass representation for minimal surfaces in Euclidean space provides a method to explicitly determine the Christoffel dual of a tri-axial ellipsoid, so do Kobayashi’s and Konderak’s Weierstrass type representations for maximal and timelike minimal surfaces in Minkowski space to find the Christoffel duals of hyperboloids, see [10] resp [11]. To investigate timelike minimal surfaces we derive some results on para-holomorphic functions, in particular, we introduce para-complex analogues of the Jacobi elliptic functions in Def & Cor A.2, cf. [4].

As we work in Minkowski space, we obtain isothermic surfaces that change causal type by affine transformations: an interesting feature of these surfaces is that the lines of separation between the space- and timelike parts of such a surface follows the curvature line net, see Lemma 2.2. Indeed, part of our investigations is independent of the existence of and relation to a zero mean curvature surface: we obtain explicit representations of Christoffel duals in Euclidean as well as Minkowski ambient geometries, see Cor 3.3, Cor 4.2, Cor 4.3, Cor 5.1 and Cor 5.2.

Though we restrict ourselves to quadrics in Minkowski space that are aligned to the timelike axis of the ambient space, this restriction is not essential: the same methods will lead to similar results if a surface is in a more general position as the nature of the occurring differential equation will not change—however, computations and formulas will be less transparent.

## The ellipsoid

To set the scene we discuss ellipsoids in Euclidean space, cf [8, §5.2.21]: as we wish to establish a relation with minimal surfaces, we seek a curvature line parametrization in terms of a meromorphic function, that is, in terms of a complex variable.

To this end we adopt a new method to determine a suitable curvature line parametrization of an ellipsoid, based on two elementary observations:

- curvature line parameters (*u*, *v*) of a surface $x:\Sigma \rightarrow {\mathbb R}^3$ can be characterized as orthogonal conjugate parameters, and
- the notion of conjugate parameters is an affine notion, in particular, independent of a choice of an ambient metric and invariant under affine transformations of ${\mathbb R}^3$.

If (., .) now denotes a non-degenerate inner product on ${\mathbb R}^3$ then $(x,x)\equiv \mathrm{const}$ implies that $x\perp dx$, hence

$$\begin{aligned} 0 = (x,x_v)_u = (x_u,x_v) + (x,x_{uv}), \end{aligned}$$

that is, (*u*, *v*) are conjugate parameters if and only if they are orthogonal.

In particular, conjugate parameters of the standard round sphere $S^2\subset {\mathbb R}^3$ can be characterized by orthogonality with respect to the induced metric. Clearly, these also qualify as “curvature line parameters”, i.e., orthogonal conjugate parameters, on $S^2$.

In order to obtain curvature line coordinates on an ellipsoid we parametrize the 2-sphere conformally over a Riemann surface $\Sigma$ and post-compose by an affine transformation, more specifically,

$$\begin{aligned} \alpha x:\Sigma \rightarrow {\mathbb R}^3, \;{\mathrm{where}}\; \left\{ \begin{array}{ll} x:\Sigma \rightarrow S^2 &{} {\mathrm{is\, conformal\, and}} \\ \alpha :{\mathbb R}^3\rightarrow {\mathbb R}^3 &{} {\mathrm{scales\, the\, axes\, by }}\, a,b,c>0. \\ \end{array}\right. \end{aligned}$$

Writing *x* in terms of a meromorphic function $y:\Sigma \rightarrow {\mathbb C}\cup \{\infty \}$ we seek conditions on *y* so that, in terms of suitable holomorphic coordinates $z=u+iv:\Sigma \rightarrow {\mathbb C}$ on the Riemann surface $\Sigma$,

2.1 $$\begin{aligned} \alpha x = {1\over 1+|y|^2}\, \left( \begin{array}{c} 2a\mathop {\mathrm{Re}}y\\ 2b\mathop {\mathrm{Im}}y\\ c(1-|y|^2)\\ \end{array}\right) = {1\over 1+y{\bar{y}}}\, \left( \begin{array}{c} a(y+{\bar{y}})\\ -ib(y-{\bar{y}}) \\ c(1-y{\bar{y}}) \\ \end{array}\right) : \Sigma \rightarrow {\mathbb R}^3 \subset {\mathbb C}^3\nonumber \\ \end{aligned}$$

yields an orthogonal, hence curvature line parametrization. In terms of $z=u+iv$, orthogonality of the parameter lines $v=\mathrm{const}$ and $u=\mathrm{const}$ is expressed by the fact that the *z*-derivative

$$\begin{aligned} \mathop {\mathrm{Im}}\left( (\alpha x)',(\alpha x)'\right) = 0, \;{\mathrm{where}}\; (\alpha x)' = (\alpha x)_z = {1\over 2}\left( (\alpha x)_u - i(\alpha x)_v \right) \end{aligned}$$

and $(.,.):{\mathbb C}^3\times {\mathbb C}^3\rightarrow {\mathbb C}$ denotes the bilinear extension of the standard inner product of ${\mathbb R}^3$ to ${\mathbb C}^3$. Excluding the case $a=b=c$, when the derivative

$$\begin{aligned} (\alpha x)' = {y'\over \left( 1+y{\bar{y}}\right) ^2}\, \left( \begin{array}{c} a\,\left( 1-{\bar{y}}^2\right) \\ -ib\,\left( 1+{\bar{y}}^2\right) \\ -2c{\bar{y}}\\ \end{array}\right) \end{aligned}$$

becomes isotropic, i.e., $z\mapsto (\alpha x)(z)$ conformal, the condition that $z\mapsto ((\alpha x)',(\alpha x)')(z)\in {\mathbb R}$ be real-valued reads

2.2 $$\begin{aligned} y'^2 = \varrho \,\left\{ a^2\left( 1-y^2\right) ^2 - b^2\left( 1+y^2\right) ^2 + 4c^2y^2 \right\} , \end{aligned}$$

where $z\mapsto \varrho (z)\in {\mathbb R}$ is a suitable real-valued and holomorphic function, hence a (real) constant by the Cauchy–Riemann equations $\varrho _u+i\varrho _v=0$.

Thus the function $y:\Sigma \rightarrow {\mathbb C}\cup \{\infty \}$ is an elliptic function, defined on a suitable torus $\Sigma ={\mathbb C}/\Gamma$, and *z* can be considered as a globally defined coordinate function. For a tri-axial ellipsoid, where *a*, *b* and *c* are pairwise distinct, the four branch values

$$\begin{aligned} y' = 0 \;\Leftrightarrow \; y^2 = {1\over a^2-b^2}\left( \sqrt{a^2-c^2}\pm \sqrt{b^2-c^2}\right) ^2 \end{aligned}$$

of the elliptic function *y* yield the singularities of the curvature line net, that is, the four umbilics of the ellipsoid. Note that the set of branch values is symmetric with respect to reflections in the real and imaginary axes, we well as with respect to inversion in the unit circle. Hence, depending on the order of half-axis lengths, the branch values are all real, all purely imaginary or all unitary.

For example, for a tri-axial ellipsoid in ${\mathbb R}^3$ we may assume that $a>b>c$, without loss of generality. We then set

$$\begin{aligned} p := \sqrt{a^2-b^2\over a^2-c^2}, \quad q := \sqrt{b^2-c^2\over a^2-c^2} = \sqrt{1-p^2} \quad {\mathrm{and}}\; r := {\sqrt{a^2-c^2}\over b}; \end{aligned}$$

hence (2.2) reads

$$\begin{aligned} y'^2 = \varrho \,b^2r^2p^2\, \left( y^2-\left( {1+q\over p}\right) ^2\right) \left( y^2-\left( {1-q\over p}\right) ^2\right) . \end{aligned}$$

Then the branch values $y^2=({1\pm q\over p})^2$ of *y* are all real and symmetric with respect to the unit circle: this reflects the position and symmetry of the umbilics on such a tri-axial ellipsoid, which lie on the “equator” ellipse in the plane orthogonal to its middle length axis. As a constant real factor may be absorbed by a (constant) scaling in the domain we may, without loss of generality, assume that $1=4\varrho \,b^2r^2$ to obtain a solution of (2.2):

$$\begin{aligned} y = {1\over i}\,e^{i\mathop {\mathrm{am}}\nolimits _p} = \mathop {\mathrm{sn}}\nolimits _{p} - i\mathop {\mathrm{cn}}\nolimits _p, \;{\mathrm{where}}\; \mathop {\mathrm{am}}\nolimits _p:{\mathbb C}\rightarrow {\mathbb C}\cup \{\infty \} \end{aligned}$$

denotes the Jacobi amplitude function with module *p*, i.e., $\mathop {\mathrm{am}}\nolimits _p'=\mathop {\mathrm{dn}}\nolimits _p=\sqrt{1-p^2\mathop {\mathrm{sn}}\nolimits _p^2}$. Hence a curvature line parametrization can be expressed explicitly in terms of Jacobi elliptic functions:

### Lemma 2.1

Let $p,q\in (0,1)$ so that $p^2+q^2=1$ and $r\in (0,{1\over q})$; a curvature line parametrization of a tri-axial ellipsoid is then obtained, using the Jacobi elliptic functions $\mathop {\mathrm{sn}}\nolimits _p,\mathop {\mathrm{cn}}\nolimits _p:{\mathbb C}\rightarrow {\mathbb C}\cup \{\infty \}$ with module *p*, by

$$\begin{aligned} u+iv=z\mapsto (\alpha x)(z) : = \left( \begin{array}{c} a\mathop {\mathrm{sn}}\nolimits _pu\mathop {\mathrm{dn}}\nolimits _qv \\ -b\mathop {\mathrm{cn}}\nolimits _pu\mathop {\mathrm{cn}}\nolimits _qv\\ c\mathop {\mathrm{dn}}\nolimits _pu\mathop {\mathrm{sn}}\nolimits _qv\\ \end{array}\right) , \quad {\mathrm{where}}\; \left\{ \begin{array}{l} a := \sqrt{1+r^2p^2},\\ b := 1,\\ c := \sqrt{1-r^2q^2}. \end{array}\right. \end{aligned}$$

Conversely, up to homothety every tri-axial ellipsoid, with half-axes $a>b>c$, admits such a curvature line parametrization.

**Fig. 1:**
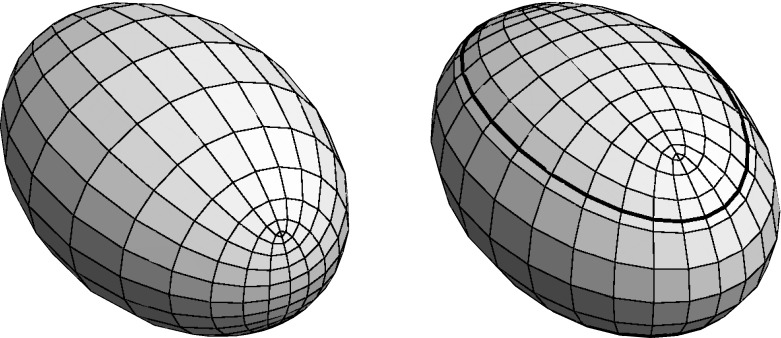
A tri-axial ellipsoid with its curvature line net in ${\mathbb R}^3$ resp ${\mathbb R}^{2,1}$

Note that the same arguments apply for a Minkowski ambient geometry: the notion of a conjugate net is independent of metric, hence only the orthogonality condition (2.2) changes by a sign—replacing *c* by *ic* in (2.2) we obtain

2.3 $$\begin{aligned} y'^2 = \varrho \,\left\{ a^2\left( 1-y^2\right) ^2 - b^2\left( 1+y^2\right) ^2 - 4c^2y^2 \right\} \end{aligned}$$

as the condition for $z=u+iv$ to yield a curvature line net for the ellipsoid parametrized by (2.1).

However, there is now a distinguished (timelike) direction, and a detailed analysis depends on how the order of half-axes lengths of the ellipsoid relates to the timelike direction. In particular, the branch values of *y* are now either all real or all purely imaginary:

$$\begin{aligned} y' = 0 \;\Leftrightarrow \; y^2 = {1\over a^2-b^2}\left( \sqrt{a^2+c^2}\pm \sqrt{b^2+c^2}\right) ^2. \end{aligned}$$

Also, note that an ellipsoid in Minkowski space decomposes into three connected components, two of which carry a positive definite induced metric while the induced metric has Lorentz signature on the remaining component—and it degenerates on the two curves separating these components, cf. Fig. 1. The umbilics necessarily lie in the spacelike part of the ellipsoid, as is confirmed by determining the points where the equator ellipse containing the umbilics intersects the curve that separates the space- and timelike parts of the ellipsoid: when $a>b$, without loss of generality, these are given by

$$\begin{aligned} y^2 = {1\over a^2}\,\left( \sqrt{a^2+c^2}\pm c\right) ^2; \end{aligned}$$

the assertion then is a consequence of the chain of inequalities

$$\begin{aligned}&\left( {\sqrt{a^2+c^2}-\sqrt{b^2+c^2}\over \sqrt{a^2-b^2}}\right) ^2< \left( {\sqrt{a^2+c^2}-c\over a}\right) ^2< 1 \\&\quad< \left( {\sqrt{a^2+c^2}+c\over a}\right) ^2 < \left( {\sqrt{a^2+c^2}+\sqrt{b^2+c^2}\over \sqrt{a^2-b^2}}\right) ^2. \end{aligned}$$

These curves of separation between the spacelike and timelike parts of the ellipsoid follow the curvature line net, hence may be considered as curvature lines as well: if (*u*, *v*) are orthogonal conjugate parameters of a surface *x*, that is,

$$\begin{aligned} x_{uv} = \lambda x_u + \mu x_v \;{\mathrm{and}}\; (x_u,x_v) = 0, \end{aligned}$$

then

$$\begin{aligned} G_u = (x_v,x_v)_u = 2\mu (x_v,x_v) = 2\mu \,G \end{aligned}$$

satisfies a first order linear differential equation; consequently, the function $u\mapsto G(u,v)$ vanishes identically if it vanishes for some *u*. Clearly, an analogous statement holds true for *E*. Thus we have proved:

### Lemma 2.2

Let $(u,v)\mapsto x(u,v)\in {\mathbb R}^{2,1}$ denote an orthogonal and conjugate parametrization of a surface patch in Minkowski space. If the induced metric degenerates at a point *x*(*u*, *v*) then it degenerates along a parameter curve through this point.

## Scherk’s saddle tower

We shall now see how the elliptic functions $y=\mathop {\mathrm{sn}}\nolimits _p-i\mathop {\mathrm{cn}}\nolimits _p$, derived above to parametrize ellipsoids by curvature lines, also yields (curvature line) parametrizations of Scherk’s singly periodic saddle towers, cf [16], see also [9, §2.3.4] and [14, §83(41)]. Moreover, the Christoffel dual of a tri-axial ellipsoid as an isothermic surface, cf. [15] or [8, §5.2.21], can be obtained from a suitable saddle tower by an affine transformation.

Given a meromorphic function $y:\Sigma \rightarrow {\mathbb C}\cup \{\infty \}$ and a polarization ${\mathfrak q}$, i.e., a meromorphic quadratic differential, on the Riemann surface $\Sigma$ the Weierstrass representation formula yields a minimal surface

3.1 $$\begin{aligned} x^*= & {} \mathop {\mathrm{Re}}\int \left( \begin{array}{c} 1-y^2\\ i(1+y^2)\\ -2y\\ \end{array}\right) \,{{\mathfrak q}\over dy} \;\mathrm{with}\;\nonumber \\ x= & {} {1\over 1+|y|^2}\, \left( \begin{array}{c} 2\mathop {\mathrm{Re}}y \\ 2\mathop {\mathrm{Im}}y \\ 1-|y|^2 \\ \end{array}\right) = {1\over 1+y{\bar{y}}}\, \left( \begin{array}{c} y+{\bar{y}} \\ {1\over i}(y-{\bar{y}})\\ 1-y{\bar{y}}\\ \end{array}\right) \end{aligned}$$

as its Gauss map and ${\mathfrak q}=(x^*_z,x_z)\,dz^2$ as its Hopf differential since

3.2 $$\begin{aligned} x^*_zdz = {{\mathfrak q}\over (x_{\bar{z}},x_z)\,dz}\,x_{\bar{z}}. \end{aligned}$$

Note that in case ${\mathfrak q}=dz^2$, that is, when $z=u+iv$ are conformal curvature line coordinates, the Weierstrass formula (3.2) simplifies to the Christoffel formula for the dual of an isothermic surface, see [2, IV] or [8, §5.2.1], cf [8, §5.3.12]:

$$\begin{aligned} x^*_z = {1\over 2y'}\, \left( \begin{array}{c} 1-y^2\\ i(1+y^2)\\ -2y\\ \end{array}\right) = {1\over (x_{\bar{z}},x_z)}\,x_{\bar{z}} \;\Leftrightarrow \; \left\{ \begin{array}{l} x^*_u ={2\over |x_u|^2}\,x_u, \\ x^*_v =-{2\over |x_v|^2}\,x_v. \\ \end{array}\right. \end{aligned}$$

When $y:\Sigma \rightarrow {\mathbb C}\cup \{\infty \}$ is an elliptic function, defined on a suitable torus $\Sigma ={\mathbb C}/\Gamma$, it is straightforward to integrate the Weierstrass formula (3.1) using partial fractions. In particular, for the function

3.3 $$\begin{aligned} y={1\over i}\,e^{i\mathop {\mathrm{am}}\nolimits _p}=\mathop {\mathrm{sn}}\nolimits _p-i\mathop {\mathrm{cn}}\nolimits _p, \;{\mathrm{satisfying}}\; y'^2 = {1\over 4}\left\{ p^2\left( 1+y^4\right) -2\left( 1+q^2\right) \,y^2 \right\} ,\nonumber \\ \end{aligned}$$

that we used in Lemma 2.1 above to obtain a curvature line parametrization of a tri-axial ellipsoid we find

3.4 $$\begin{aligned} \begin{array}{llll} \int {1-y^2\over y'^2}\,dy &{}=&{}{2\over p}\,\mathop {\mathrm{artanh}}{2y\over p(1+y^2)}, &{}\\ i\,\int {1+y^2\over y'^2}\,dy &{}=&{} {2i\over pq}\mathop {\mathrm{artanh}}{2qy\over p(1-y^2)} &{}\quad =\;\; {2\over pq}\,\arctan {2iqy\over p(1-y^2)}, \\ \int {2y\over y'^2}\,dy &{}=&{}{2\over q}\,\mathop {\mathrm{artanh}}{q(1+y^2)\over (1-y^2)}. &{} \end{array} \end{aligned}$$

Hence we learn (cf. “Appendix”) that the minimal surface $x^*$ of (3.1) obtained from (3.3) has an implicit representation

3.5 $$\begin{aligned} q^2\cosh px^*_1 + \cos pqx^*_2 - p^2\cosh qx^*_3 = 0, \end{aligned}$$

thus it yields a curvature line parametrization of a Scherk tower, cf. [16, §8] and [14, §83(41)]:

### Lemma 3.1

Let $p,q\in (0,1)$ so that $p^2+q^2=1$; a curvature line parametrization of a Scherk tower is then obtained, using the Jacobi elliptic functions $\mathop {\mathrm{sn}}\nolimits _p,\mathop {\mathrm{cn}}\nolimits _p,\mathop {\mathrm{dn}}\nolimits _p:{\mathbb C}\rightarrow {\mathbb C}\cup \{\infty \}$ with module *p*, by

$$\begin{aligned} z\mapsto x^*(z) := {2\over pq}\mathop {\mathrm{Re}}\, \left( \begin{array}{c} q\mathop {\mathrm{artanh}}{1\over p\mathop {\mathrm{sn}}\nolimits _p}\\ \phantom {q}\arctan {q\over p\mathop {\mathrm{cn}}\nolimits _p}\\ p\mathop {\mathrm{artanh}}{q\over i}{\mathop {\mathrm{sn}}\nolimits _p\over \mathop {\mathrm{cn}}\nolimits _p} \end{array}\right) (z). \end{aligned}$$

Thus considering $(x,x^*)$ as a Christoffel pair, any affine image of the pair will form a Combescure pair: under any affine transformation $\alpha :{\mathbb R}^3\rightarrow {\mathbb R}^3$ the common (conformal) curvature line parameters (*u*, *v*) of *x* and $x^*$ are turned into common conjugate parameters of $\alpha x$ and $\alpha x^*$, with parallel tangents [for conciseness, we drop parentheses in $\alpha x_{\bar{z}}=(\alpha x)_{\bar{z}}=d\alpha (x_{\bar{z}})$],

$$\begin{aligned} x^*_z = {1\over (x_z,x_{\bar{z}})}\,x_{\bar{z}} \;\Rightarrow \; \alpha x^*_z = {1\over (x_z,x_{\bar{z}})}\,\alpha x_{\bar{z}}. \end{aligned}$$

Then $\alpha x$ and $\alpha x^*$ are conformally related if and only if (*u*, *v*) are orthogonal coordinates, that is, curvature line coordinates for both surfaces. Namely, if $\alpha x^*_z=\varrho \,\alpha x_{\bar{z}}$ with a (real) function $\varrho$, then

$$\begin{aligned}&|\alpha x^*_u|^2du^2 + 2\left( \alpha x^*_u,\alpha x^*_v\right) \,dudv + |\alpha x^*_u|^2du^2\\&\quad = \varrho ^2\left\{ |\alpha x_u|^2du^2 - 2\left( \alpha x_u,\alpha x_v\right) \,dudv + |\alpha x_u|^2du^2 \right\} . \end{aligned}$$

In fact, we obtain a stronger result, as integrability of $dx^*$ implies that $z=u+iv$ yield conjugate parameters, since

$$\begin{aligned} 0 = x^*_{uv} - x^*_{vu} = (\varrho x_u)_v + (\varrho x_v)_u = 2\varrho x_{uv} + \varrho _vx_u + \varrho _ux_v. \end{aligned}$$

Thus, using Christoffel’s characterization [2] of a Christoffel pair of isothermic surfaces as an orientation reversing conformal Combescure pair, cf. [13, Main Thm] or [12, Prop 3.1], we obtain the following

### Lemma 3.2

Suppose that the differentials of two surfaces *x* and $x^*$ in ${\mathbb R}^3$ are related by

$$\begin{aligned} x^*_z = \varrho \,x_{\bar{z}} \;\Leftrightarrow \; \left\{ \begin{array}{l} x^*_u = \varrho \,x_u, \\ x^*_v = -\varrho \,x_v. \\ \end{array}\right. \end{aligned}$$

Then (*u*, *v*) are common conjugate parameters for *x* and $x^*$. Moreover, if (*u*, *v*) are orthogonal coordinates for some ambient metric (., .),

$$\begin{aligned} x_u\perp x_v \;\Leftrightarrow \; z\mapsto (x_z,x_z)(z)\in {\mathbb R}, \end{aligned}$$

then $(x,x^*)$ is a Christoffel pair with common curvature line parameters (*u*, *v*).

Note that, starting as above with common conformal curvature line coordinates (*u*, *v*) of a Christoffel pair $(x,x^*)$ to obtain a new Christoffel pair $(\alpha x,\alpha x^*)$ by an affine transformation via the orthogonality condition of Lemma 3.2, the coordinates will in general not be conformal for either surface, $\alpha x$ or $\alpha x^*$: in general $(\alpha x_z,\alpha x_z)$ does not vanish, even if it is real. However, as *x* satisfies a Laplace equation

$$\begin{aligned} x_{uv} = \lambda x_u + \mu x_v \end{aligned}$$

when (*u*, *v*) are conjugate parameters, we infer that

$$\begin{aligned} {1\over 2}\left( \ln {(\alpha x_u,\alpha x_u)\over (x_u,x_u)}\right) _v = \mu \,\left\{ {(\alpha x_u,\alpha x_v)\over (\alpha x_u,\alpha x_u)} - {(x_u,x_v)\over (x_u,x_u)} \right\} = 0 \;\mathrm{and}\; {1\over 2}\left( \ln {(\alpha x_v,\alpha x_v)\over (x_v,x_v)}\right) _u = 0 \end{aligned}$$

as soon as (*u*, *v*) are curvature line coordinates for both, *x* and $\alpha x$. Consequently, conformal curvature line coordinates $(\tilde{u},\tilde{v})$ for $\alpha x$ are obtained from those of *x* by integrating

$$\begin{aligned} d\tilde{u} = {|\alpha x_u|\over |x_u|}\,du \;\mathrm{and}\; d\tilde{v} = {|\alpha x_v|\over |x_v|}\,dv. \end{aligned}$$

Identification of conformal curvature line parameters for $\alpha x$ is more involved if a reference conformal factor is missing, that is, if (*u*, *v*) were not conformal curvature line parameters for *x*.

Combining the results from Lemmas 2.1, 3.1 and 3.2 we learn how a solution *y* of (2.2) gives rise to a curvature line parametrization of a tri-axial ellipsoid on the one hand, and to a curvature line parametrization of a Scherk tower on the other hand—hence, by Lemma 3.2, to a curvature line parametrization of the Christoffel dual of the tri-axial ellipsoid, cf. [15, §6]:

### Cor 3.3

Let $p,q\in (0,1)$ so that $p^2+q^2=1$ and $r\in (0,{1\over q})$; the Christoffel dual of the tri-axial ellipsoid with half axes

$$\begin{aligned} a := \sqrt{1+r^2p^2}, \; b := 1 \;\mathrm{and}\; c := \sqrt{1-r^2q^2} \end{aligned}$$

is an affine transformation of a Scherk tower, thus in terms of Jacobi elliptic functions given by

$$\begin{aligned} z\mapsto \alpha x^*(z) := \mathop {\mathrm{Re}}\, \left( \begin{array}{c} {2a\over p}\mathop {\mathrm{artanh}}{1\over p\mathop {\mathrm{sn}}\nolimits _p}\\ {2b\over pq}\arctan {q\over p\mathop {\mathrm{cn}}\nolimits _p}\\ {2c\over q}\mathop {\mathrm{artanh}}{q\over i}{\mathop {\mathrm{sn}}\nolimits _p\over \mathop {\mathrm{cn}}\nolimits _p} \end{array}\right) (z). \end{aligned}$$

**Fig. 2:**
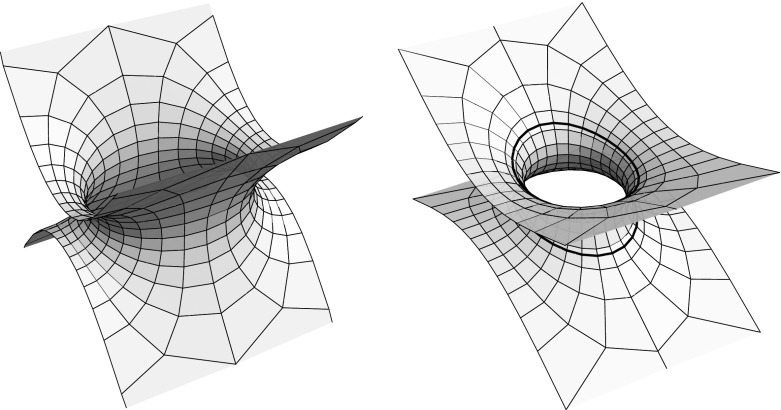
The Christoffel duals of the ellipsoids in ${\mathbb R}^3$ resp ${\mathbb R}^{2,1}$ of Fig. 1

Lemma 3.2 holds also for spacelike or timelike real isothermic surfaces in a Minkowski ambient geometry: apart from the characterization of curvature line coordinates as orthogonal conjugate parameters the proof of Lemma 3.2 only relies on Christoffel’s formula

$$\begin{aligned} x^*_z = {1\over (x_z,x_{\bar{z}})}\,x_{\bar{z}}, \end{aligned}$$

which holds in both the spacelike and timelike real isothermic cases, cf. [13, Main Thm]. Hence we obtain a Christoffel dual for a tri-axial ellipsoid in Minkowski space, see Fig. 2.

However, the standard 2-sphere $S^2\subset {\mathbb R}^3$ is not totally umbilic in ${\mathbb R}^{2,1}$, hence inverse stereographic projection of a holomorphic function *y* does in general not yield an orthogonal net on $S^2$. In particular, the solution *y* of (2.3) that provides a curvature line net of a tri-axial ellipsoid by inverse stereographic projection followed by a suitable affine transformation does in general not yield a curvature line net on the 2-sphere, before the affine transformation: setting $a=b$ in (2.3) confirms that a curvature line net of $S^2$ as a non-umbilic surface of revolution in ${\mathbb R}^{2,1}$ is necessarily given by its meridians and circles of latitude.

Note that the catenoid is still the Christoffel dual of the (Euclidean) sphere as a surface of revolution in Minkowski space, since the meridians and lines of latitude are orthogonal: the Christoffel dual $(u,v)\mapsto x^*(u,v)$ of a surface of revolution $(u,v)\mapsto x(u,v)$ can explicitly be computed as

$$\begin{aligned} x^*(u,v) = \left( \begin{array}{c} -{1\over r(u)}\cos v\\ -{1\over r(u)}\sin v\\ \int {h'(u)du\over r^2(u)} \end{array}\right) , \;\mathrm{where}\; x(u,v) = \left( \begin{array}{c} r(u)\cos v\\ r(u)\sin v\\ h(u)\\ \end{array}\right) , \end{aligned}$$

since

$$\begin{aligned} x^*_z(u,v) = {1\over r^2(u)}\,x_{\bar{z}}(u,v) \;\mathrm{and}\; \left( x_z(u,v),x_z(u,v)\right) = {\left( r'^2\pm h'^2\right) -r^2\over 4}(u) \in {\mathbb R}\end{aligned}$$

for the standard metrics (., .) of signature $(+,+,\pm )$ on ${\mathbb R}^3$. Hence Lemma 3.2 shows that the Minkowski Christoffel dual of a surface of revolution with timelike axis coincides with its Euclidean dual. In particular, for $r(u)={1\over \cosh u}$ and $h(u)=\tanh u$, the conformal curvature line parametrizations of the catenoid and its Gauss map are obtained.

## The spacelike hyperboloid

In order to obtain a similar relation between the Christoffel duality of a quadric and an affine transformation of a zero mean curvature surface in Minkowski space, as we did for the Christoffel dual of an ellipsoid in Euclidean space in the previous section, we will need to focus on a totally umbilic quadric in ${\mathbb R}^{2,1}$. As we shall see, the same methodology then yields similar results.

Thus consider inverse stereographic projection onto the standard 2-sheeted hyperboloid in ${\mathbb R}^{2,1}$ as a conformal parametrization of the hyperboloid:

$$\begin{aligned} \left( {\mathbb C}\cup \{\infty \}\right) {\setminus }S^1\ni y \mapsto x:= & {} {1\over 1-|y|^2}\, \left( \begin{array}{c} 2\mathop {\mathrm{Re}}y\\ 2\mathop {\mathrm{Im}}y\\ 1+|y|^2\\ \end{array}\right) = {1\over 1-y{\bar{y}}}\, \left( \begin{array}{c} (y+{\bar{y}}) \\ -i(y-{\bar{y}}) \\ 1+y{\bar{y}} \\ \end{array}\right) \\\in & {} {\mathbb R}^{2,1} \subset {\mathbb C}^3. \end{aligned}$$

A parametrization of a general 2-sheeted hyperboloid, with timelike principal axis, is then obtained as as an affine transformation of the standard hyperboloid,

4.1 $$\begin{aligned} \alpha x = {1\over 1-|y|^2}\, \left( \begin{array}{c} 2a\mathop {\mathrm{Re}}y\\ 2b\mathop {\mathrm{Im}}y\\ c(1+|y|^2)\\ \end{array}\right) = {1\over 1-y{\bar{y}}}\, \left( \begin{array}{c} a(y+{\bar{y}}) \\ -ib(y-{\bar{y}}) \\ c(1+y{\bar{y}}) \\ \end{array}\right) : \Sigma \rightarrow {\mathbb R}^{2,1} \subset {\mathbb C}^3.\nonumber \\ \end{aligned}$$

As before, the orthogonality condition $z\mapsto ((\alpha x)',(\alpha x)')(z)\in {\mathbb R}$ for the conjugate parameters (*u*, *v*), written as a complex parameter $z=u+iv$, yields an elliptic differential equation

**Fig. 3:**
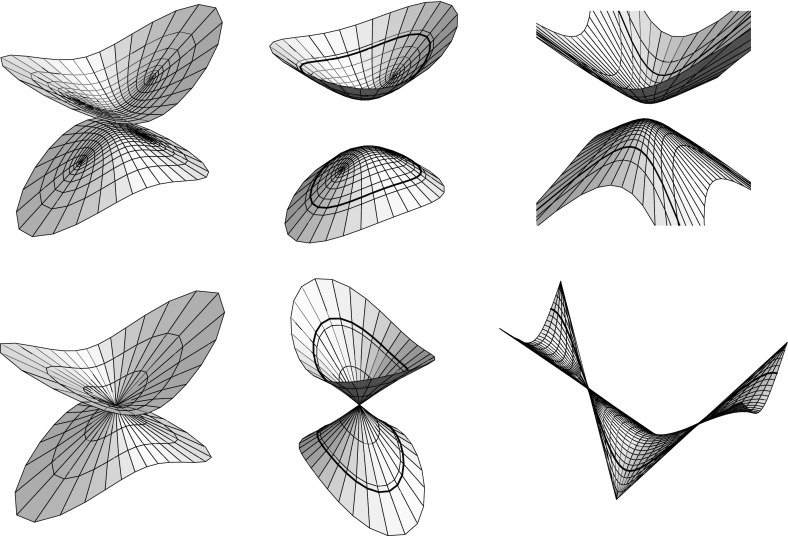
Three types of 2-sheeted hyperboloids and their Christoffel duals in ${\mathbb R}^{2,1}$

4.2 $$\begin{aligned} y'^2 = \varrho \,\left\{ a^2\left( 1+y^2\right) ^2 - b^2\left( 1-y^2\right) ^2 - 4c^2y^2 \right\} , \end{aligned}$$

with its branch values

$$\begin{aligned} y^2 = -{1\over a^2-b^2}\left( \sqrt{a^2-c^2}\pm \sqrt{b^2-c^2}\right) ^2 \end{aligned}$$

determining the umbilics of the 2-sheeted hyperboloid in Minkowski space ${\mathbb R}^{2,1}$. Note that (4.2) may be obtained from the equation (2.2) for a Euclidean ellipsoid by replacing *y* by *iy* and a sign change of $\varrho$. However, in contrast to the case of an ellipsoid in Euclidean space, the hyperboloid has a distinguished (timelike) axis and various geometric configurations may occur, as an elementary case study reveals.

(i) $a,b\ge c$: in this case, the 2-sheeted hyperboloid is spacelike and, as long as the half-axis lengths are pairwise distinct, the branch values of *y* are either real or imaginary and yield the four umbilics of the 2-sheeted hyperboloid, with their symmetries reflected by the symmetries of the branch values of *y*, as in the case of the ellipsoid, cf. Fig. 3, left. If $a=b$ the hyperboloid is a surface of revolution with only two umbilics, given by branch values at $y=0$ and $y=\infty$. If, on the other hand, $a=c$ or $b=c$ then the branch values of *y* become unitary, reflecting the absence of umbilics in this case.
(ii) $a,b<c$: now the hyperboloid has mixed causal type, each component consists of a (compact) spacelike part and an (annular) timelike part. Unless $a=b$ and we obtain a surface of revolution, the hyperboloid has four umbilics in its spacelike part, given by the real or imaginary branch values of *y*, cf. Fig. 3, middle. Note that, by Lemma 2.2, the lines where the causal type of the hyperboloid changes are aligned with the curvature line net.
(iii) $a<c\le b$ or $a\ge c>b$: in these cases, the hyperboloid has also mixed causal type, but now both components decompose into one spacelike and two timelike parts, cf. Fig. 3, right, and the branch values of *y* become unitary, reflecting the fact that these hyperboloids have no umbilics.

Thus, in analogy to Lemma 2.1 for curvature line parameters of Euclidean ellipsoids, we obtain curvature line parameters for 2-sheeted hyperboloids in Minkowski space in terms of Jacobi elliptic functions, based on the same methods as in the ellipsoid case:

### Lemma 4.1

Any tri-axial 2-sheeted hyperboloid in Minkowski space admits, up to homothety, a curvature line parametrization using the Jacobi elliptic functions $\mathop {\mathrm{cn}}\nolimits _p,\mathop {\mathrm{sn}}\nolimits _p:{\mathbb C}\rightarrow {\mathbb C}\cup \{\infty \}$,

$$\begin{aligned} u+iv=z\mapsto \alpha x(z) := {1\over \mathop {\mathrm{dn}}\nolimits _pu\mathop {\mathrm{sn}}\nolimits _qv}\, \left( \begin{array}{l} a\mathop {\mathrm{cn}}\nolimits _pu\mathop {\mathrm{cn}}\nolimits _qv \\ b\mathop {\mathrm{sn}}\nolimits _pu\mathop {\mathrm{dn}}\nolimits _qv \\ c \\ \end{array}\right) , \;\mathrm{where}\; \left\{ \begin{array}{l} a := \sqrt{1+r^2p^2},\\ b := 1,\\ c := \sqrt{1-r^2q^2}\\ \end{array}\right. \end{aligned}$$

and $p,q,r\in {\mathbb R}\cup i{\mathbb R}$ with $p^2+q^2=1$ satisfy one of the following:

$$\begin{aligned}&\mathrm{(i)}\; p,q\in (0,1), r\in \left( 0,{1\over q}\right) ; \quad \\&\mathrm{(ii)}\; p\in i{\mathbb R}, q>1, {r\over i}\in \left( 0,{1\over q}\right) ; \\&\mathrm{(iii)}\; p>1, q\in i{\mathbb R}, {r\over i}\in \left( 0,{1\over p}\right) . \end{aligned}$$

As Lemma 3.2, which served as the key step in characterizing the Christoffel dual of a Euclidean ellipsoid as an affine image of a Scherk saddle tower, did not depend on the signature of the ambient metric an analogous result is obtained for the Christoffel duals of 2-sheeted hyperboloids in Minkowski space, cf Cor 3.3.

Employing the Weierstrass representation of [10, Thm 1.1] for maximal surfaces in Minkowski space,

4.3 $$\begin{aligned} x^*= \mathop {\mathrm{Re}}\int \left( \begin{array}{c} 1+y^2\\ i\,\left( 1-y^2\right) \\ 2y\\ \end{array}\right) \,{{\mathfrak q}\over dy} \;\mathrm{with}\; x = {1\over 1-y{\bar{y}}}\, \left( \begin{array}{c} y+{\bar{y}} \\ {1\over i}\,\left( y-{\bar{y}}\right) \\ 1+y{\bar{y}} \\ \end{array}\right) \end{aligned}$$

as the Gauss map and ${\mathfrak q}=dz^2$ as a polarization of the (universal cover of the) underlying Riemann surface $\Sigma ={\mathbb C}$ yields again Christoffel’s formula for the dual of a (spacelike) isothermic surface,

$$\begin{aligned} x^*_z = {1\over 2y'}\, \left( \begin{array}{c} 1+y^2\\ i\,\left( 1-y^2\right) \\ 2y\\ \end{array}\right) = {1\over \left( x_{\bar{z}},x_z\right) }\,x_{\bar{z}} \;\Leftrightarrow \; \left\{ \begin{array}{c} x^*_u = {2\over |x_u|^2}\,x_u, \\ x^*_v =-{2\over |x_v|^2}\,x_v. \end{array}\right. \end{aligned}$$

Thus replacing *y* by *iy* in (3.3), to obtain a solution of (4.2) with $\varrho =-{1\over 4(a^2-c^2)}$ from that of (2.2), we arrive at

4.4 $$\begin{aligned} y=e^{i\mathop {\mathrm{am}}\nolimits _p}=\mathop {\mathrm{cn}}\nolimits _p+i\mathop {\mathrm{sn}}\nolimits _p, \;\mathrm{satisfying}\; y'^2 = -{1\over 4}\left\{ p^2\left( 1+y^4\right) +2\left( 1+q^2\right) \,y^2 \right\} .\nonumber \\ \end{aligned}$$

Using (3.4) we hence integrate (4.3),

4.5 $$\begin{aligned} \int {1+y^2\over y'^2}\,dy= & {} -{2\over p}\,\arctan {2y\over p(1-y^2)}, \nonumber \\ i\int {1-y^2\over y'^2}\,dy= & {} -{2i\over pq}\arctan {2qy\over p(1+y^2)}= {2\over pq}\,\mathop {\mathrm{artanh}}{2qy\over ip(1+y^2)}, \nonumber \\ \int {2y\over y'^2}\,dy= & {} {2\over q}\,\mathop {\mathrm{artanh}}{q(1-y^2)\over (1+y^2)}. \end{aligned}$$

As the timelike $x_3$-axis is geometrically distinguished we obtain (cf. “Appendix”) two qualitatively different implicit representations of the maximal surfaces $x^*$ obtained from (4.4), cf [6, Sect 3]:

4.6 $$\begin{aligned}&q^2\cos px^*_1 + \cosh pqx^*_2 - p^2\cosh qx^*_3 = 0 \quad \mathrm{if} p<1 \;\mathrm{and}\; q\in (0,1); \nonumber \\&q^2\cos px^*_1 + \cos {pqx^*_2\over i} - p^2\cos {qx^*_3\over i} = 0 \quad \mathrm{if} p>1 \;\mathrm{and}\; q\in i{\mathbb R}. \end{aligned}$$

For the tri-axial 2-sheeted hyperboloid this yields another “permutability theorem”, intertwining Christoffel duality and affine transformation:

### Cor 4.2

The Christoffel dual of a tri-axial 2-sheeted hyperboloid in Minkowski space with half axes lengths

$$\begin{aligned} \mathrm{(i)}\; a>b>c, \quad \mathrm{(ii)}\; c>a>b \quad \mathrm{or}\quad \mathrm{(iii)}\; b>c>a \quad \end{aligned}$$

is the affine image of a maximal surface, more precisely, up to homothety it is given by

$$\begin{aligned} z\mapsto \alpha x^*(z) := \mathop {\mathrm{Re}}\, \left( \begin{array}{c} {2a\over p}\arctan {1\over ip\mathop {\mathrm{sn}}\nolimits _p}\\ {2b\over pq}\mathop {\mathrm{artanh}}{q\over ip\mathop {\mathrm{cn}}\nolimits _p}\\ {2c\over q}\mathop {\mathrm{artanh}}{q\over i}{\mathop {\mathrm{sn}}\nolimits _p\over \mathop {\mathrm{cn}}\nolimits _p} \end{array}\right) (z) \;\mathrm{with}\; \left\{ \begin{array}{l} p := \sqrt{a^2-b^2\over a^2-c^2}, \\ q := \sqrt{b^2-c^2\over a^2-c^2}. \end{array}\right. \end{aligned}$$

Note that the hyperboloid as well as its dual are spacelike in case (i), whereas both surfaces change causal type in the cases (ii) and (iii), even though the employed maximal surfaces are spacelike, cf Fig. 3. On their timelike parts the surfaces are real isothermic in the sense of [13, Sect. 2.1], that is, admit Lorentz conformal curvature line parameters, and the curvature line nets on the spacelike and timelike parts of the surfaces extend across the lines of causal type change, which follow the curvature line net by Lemma 2.2. In case (iii) the Christoffel dual surface has a translational period, cf. Fig. 3, and the surface can be extended to a triply periodic surface in Minkowski space, as is also seen from the implicit representation (4.6).

As the 2-sheeted hyperboloid is not totally umbilic in Euclidean space, the construction of the Christoffel dual as an affine image of a minimal surface fails for a hyperboloid in a Euclidean ambient geometry. However, the only failure of the construction turns out to be the minimality: integrating

$$\begin{aligned} x^*_z = {1\over 2y'}\, \left( \begin{array}{c} 1+y^2\\ i\left( 1-y^2\right) \\ 2y\\ \end{array}\right) = {2|y'|^2\over \left( 1-|y|^2\right) ^2}\,x_{\bar{z}} \;\mathrm{with}\; x = {1\over 1-|y|^2}\, \left( \begin{array}{c} 2\mathop {\mathrm{Re}}y \\ 2\mathop {\mathrm{Im}}y \\ 1+|y|^2 \\ \end{array}\right) \end{aligned}$$

we obtain $x^*$ as the (harmonic) real part of a holomorphic curve in ${\mathbb C}^3$ that is not a null curve though, hence does not yield a minimal surface $x^*$ in ${\mathbb R}^3$. As the pair $(x,x^*)$ satisfies the assumption of Lemma 3.2 we just wheel out the orthogonality condition $z\mapsto ((\alpha x)',(\alpha x)')(z)\in {\mathbb R}$ for some affine image $\alpha x$ of *x* to obtain a Christoffel pair $(\alpha x,\alpha x^*)$, with common curvature line coordinates given by a solution *y* of the elliptic differential equation

4.7 $$\begin{aligned} y'^2 = \varrho \,\left\{ a^2\left( 1+y^2\right) ^2 - b^2\left( 1-y^2\right) ^2 + 4c^2y^2 \right\} , \end{aligned}$$

with real or imaginary branch values given by

$$\begin{aligned} y^2 = -{1\over a^2-b^2}\left( \sqrt{a^2+c^2}\pm \sqrt{b^2+c^2}\right) ^2. \end{aligned}$$

**Fig. 4:**
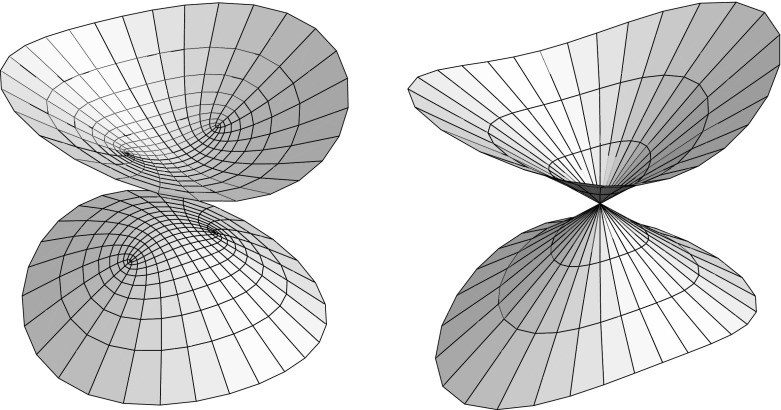
The Euclidean 2-sheeted hyperboloid and its Christoffel dual, cf. Fig. 3

As long as $a\ne b$, that is, the hyperboloid is not a surface of revolution, the four branch values are symmetric with respect to the origin as well as to the unit circle, reflecting the symmetry of the four umbilics of the 2-sheeted hyperboloid, cf. Fig. 4.

More precisely, assuming without loss of generality $a>b$ for a non-rotational 2-sheeted hyperboloid, and with $0=1+4\varrho (a^2+c^2)$, we obtain

$$\begin{aligned} y = \mathop {\mathrm{cn}}\nolimits _p+i\mathop {\mathrm{sn}}\nolimits _p = e^{i\mathop {\mathrm{am}}\nolimits _p} \;\mathrm{with}\; p = \sqrt{a^2-b^2\over a^2+c^2} \in (0,1) \end{aligned}$$

as a solution of (4.7), completely analogous to (4.2). Consequently, a curvature line parametrization of the Christoffel dual of a 2-sheeted hyperboloid in Euclidean space is obtained by precisely the same formulas as in Minkowski space, in Cor 4.2, cf. [15, §8]:

### Cor 4.3

The Christoffel dual of a non-rotational 2-sheeted hyperboloid in Euclidean space with half axes lengths $a,b,c>0$, where $1+({x_1\over a})^2+({x_2\over b})^2=({x_3\over c})^2$ and $a>b$ without loss of generality, admits a curvature line parametrization $z\mapsto \alpha x^*(z)$ by Jacobi elliptic functions:

$$\begin{aligned} z\mapsto \alpha x^*(z) := \mathop {\mathrm{Re}}\, \left( \begin{array}{c} {2a\over p}\arctan {1\over ip\mathop {\mathrm{sn}}\nolimits _p}\\ {2b\over pq}\mathop {\mathrm{artanh}}{q\over ip\mathop {\mathrm{cn}}\nolimits _p}\\ {2c\over q}\mathop {\mathrm{artanh}}{q\over i}{\mathop {\mathrm{sn}}\nolimits _p\over \mathop {\mathrm{cn}}\nolimits _p} \end{array}\right) (z) \;\mathrm{with}\; \left\{ \begin{array}{l} p := \sqrt{a^2-b^2\over a^2+c^2}, \\ q := \sqrt{b^2+c^2\over a^2+c^2}. \end{array}\right. \end{aligned}$$

## The timelike hyperboloid

In the previous section we investigated the dual of a tri-axial 2-sheeted hyperboloid as an affine transform of a maximal surface that was obtained as a dual of the standard totally umbilic 2-sheeted hyperboloid in Minkowski space ${\mathbb R}^{2,1}$. However, the Lorentz sphere yields another totally umbilic quadric, thus provides a starting point for a similar investigation of the Christoffel transforms of 1-sheeted hyperboloids.

As the 1-sheeted hyperboloid $S^{1,1}=\{x\in {\mathbb R}^{2,1}\,|(x,x)=1\}$ is a timelike surface in Minkowski space Christoffel duality leads to Konderak’s Weierstrass-type representation [11, Thm 3.3] for minimal timelike surfaces in Minkowski space: instead of holomorphic functions this representation employs para-holomorphic functions, defined on the algebra of Lorentz-numbers,

$$\begin{aligned} {\mathbb L}= \left\{ y_1+jy_2\,|\,y_1,y_2\in {\mathbb R}\right\} , \;\mathrm{where}\; j^2 = 1, \end{aligned}$$

cf. [3] or [7]. Note that ${\mathbb L}$ can be considered as the Clifford algebra of the real line with its usual operations. More specifically, we consider the inverse stereographic projection

$$\begin{aligned} {\mathbb L}{\setminus }S^1\ni y \mapsto x : = {1\over 1-\Vert y\Vert ^2}\, \left( \begin{array}{c} 2\mathop {\mathrm{Re}}y\\ 2\mathop {\mathrm{Im}}y\\ 1+\Vert y\Vert ^2\\ \end{array}\right) = {1\over 1-y{\bar{y}}}\, \left( \begin{array}{c} y+{\bar{y}} \\ {1\over j}(y-{\bar{y}}) \\ 1+y{\bar{y}} \\ \end{array}\right) \in S^{1,1}\subset {\mathbb R}^{2,1} \end{aligned}$$

into the Minkowski space of signature $(-,+,+)$, where ${\bar{y}}$ and $\Vert y\Vert$ denote the conjugate resp the modulus of a Lorentz number $y\in {\mathbb L}$,

$$\begin{aligned} \Vert y\Vert ^2 = y{\bar{y}} = (y_1+jy_2)(y_1-jy_2) = y_1^2-y_2^2 \;\mathrm{and}\; \left\{ \begin{array}{l} \mathop {\mathrm{Re}}(y_1+jy_2) = y_1, \\ \mathop {\mathrm{Im}}(y_1+jy_2) = y_2, \\ \end{array}\right. \end{aligned}$$

as usual. With a para-holomorphic function $y:{\mathbb L}\rightarrow {\mathbb L}$, $z\mapsto y(z)$, an analogue of the Enneper–Weierstrass representation formula then yields a conformal curvature line parametrization

5.1 $$\begin{aligned} x^*= -\mathop {\mathrm{Re}}\int \left( \begin{array}{c} 1+y^2\\ j\left( 1-y^2\right) \\ 2y\\ \end{array}\right) \,{dz\over y'} \end{aligned}$$

of a timelike minimal surface with Gauss map *x*, cf. [11, Thm 3.3]: as the Christoffel formula

$$\begin{aligned} x^*_z = -{1\over 2y'}\, \left( \begin{array}{c} 1+y^2\\ j\left( 1-y^2\right) \\ 2y\\ \end{array}\right) = {1\over \left( x_{\bar{z}},x_z\right) }\,x_{\bar{z}} \;\Leftrightarrow \; \left\{ \begin{array}{l} x^*_u = {2\over |x_u|^2}\,x_u, \\ x^*_v = {2\over |x_v|^2}\,x_v \\ \end{array}\right. \end{aligned}$$

holds with $z=u+jv$, by the para-complex version of the Cauchy–Riemann equations

$$\begin{aligned} 0 = 2y_{\bar{z}} = \left( (y_1)_u-(y_2)_v\right) + j\left( (y_2)_u-(y_1)_v\right) , \end{aligned}$$

we deduce that $x\perp dx^*$ and, with Lemma 3.2, that (*u*, *v*) are conjugate parameters for $x^*$ which are orthogonal as they are conformal,

$$\begin{aligned} (x_z,x_z) = {1\over 4}\left\{ \left( |x_u|^2+|x_v|^2\right) + 2j(x_u,x_v) \right\} = 0. \end{aligned}$$

Note that $(x,x^*)$ forms a pair of *real isothermic* surfaces, in the sense of [13, Sect. 2] as the surfaces have real curvature directions: in general, the shape operator of a timelike surface in Minkowski space may have complex conjugate eigendirections, or not diagonalize at all.

Wheeling out the orthogonality condition for the coordinates of an affine transform

5.2 $$\begin{aligned} \alpha x = {1\over 1-\Vert y\Vert ^2}\, \left( \begin{array}{c} 2a\mathop {\mathrm{Re}}y\\ 2b\mathop {\mathrm{Im}}y\\ c(1+\Vert y\Vert ^2)\\ \end{array}\right) = {1\over 1-y{\bar{y}}}\, \left( \begin{array}{c} a(y+{\bar{y}}) \\ jb(y-{\bar{y}}) \\ c(1+y{\bar{y}}) \\ \end{array}\right) : \Sigma \rightarrow {\mathbb R}^{2,1} \subset {\mathbb L}^3,\qquad \end{aligned}$$

as in the spacelike cases, we arrive now at the Lorentzian ordinary differential equation

5.3 $$\begin{aligned} y'^2 = -\varrho \,\left\{ a^2\left( 1+y^2\right) ^2 - b^2\left( 1-y^2\right) ^2 - 4c^2y^2 \right\} \end{aligned}$$

with a real function $\varrho$ as the condition for $z=u+jv$ to yield a (real) curvature line net for the hyperboloid parametrized by (5.2), cf. (2.2) and (4.2). Then, similar to the complex case, the para-complex Cauchy–Riemann equations imply that the real function $\varrho$ is, in fact, a real constant, since $y_{\bar{z}}=0$ implies $\varrho _{\bar{z}}=0$.

Note though that the apparent similarity with the cases previously examined is deceptive: in contrast to the elliptic Eqs. (4.2), or (2.2), we are now dealing with a para-holomorphic function *y* and its para-complex derivative in (5.3). However, as the differential equations are real, we may employ a real solution of (4.2) to obtain a real solution of (5.3) that we may then extend to a para-holomorphic solution. To make this idea more tangible consider our above solution

$$\begin{aligned} y(z) = e^{i\mathop {\mathrm{am}}\nolimits _pz} = \mathop {\mathrm{cn}}\nolimits _pz + i\mathop {\mathrm{sn}}\nolimits _pz, \;\mathrm{where}\; p = \sqrt{a^2-b^2\over a^2-c^2}, \end{aligned}$$

of the elliptic Eq. (4.2),

$$\begin{aligned} y'^2= & {} -{1\over 4}\left\{ p^2\left( y^4+1\right) + 2\left( 1+q^2\right) \,y^2 \right\} \\= & {} -{1\over 4\left( a^2-c^2\right) } \left\{ a^2\left( 1+y^2\right) ^2 - b^2\left( 1-y^2\right) ^2 - 4c^2y^2 \right\} . \end{aligned}$$

We may then replace *y*(*z*) by *y*(*iz*) to obtain a real solution of (5.3) with a suitable choice of the constant, $\varrho =-{1\over 4(a^2-c^2)}$, by restriction of the complex solution to real variables:

5.4 $$\begin{aligned} y(z)= & {} e^{i\mathop {\mathrm{am}}\nolimits _piz} = {1-\mathop {\mathrm{sn}}\nolimits _qz\over \mathop {\mathrm{cn}}\nolimits _qz} = {\mathop {\mathrm{cn}}\nolimits _qz\over 1+\mathop {\mathrm{sn}}\nolimits _qz} \;{\mathrm{solves}}\nonumber \\ y'^2= & {} {1\over 4}\left\{ p^2\left( 1+y^4\right) + 2\left( 1+q^2\right) \,y^2 \right\} . \end{aligned}$$

Note that the conversion formulae for reciprocal and imaginary moduli do not affect reality of the Jacobi elliptic functions, hence this approach does not depend on an order of the half axes lengths.

We then extend this real analytic solution (uniquely, cf Lemma A.1) to a Lorentz-analytic solution

5.5 $$\begin{aligned} y(u+jv) = y(u+v)\,{1+j\over 2} + y(u-v)\,{1-j\over 2} = {\mathop {\mathrm{cn}}\nolimits _qu-j\mathop {\mathrm{sn}}\nolimits _qv\mathop {\mathrm{dn}}\nolimits _qu\over \mathop {\mathrm{cn}}\nolimits _qv+\mathop {\mathrm{sn}}\nolimits _qu\mathop {\mathrm{dn}}\nolimits _qv}.\quad \end{aligned}$$

**Fig. 5:**
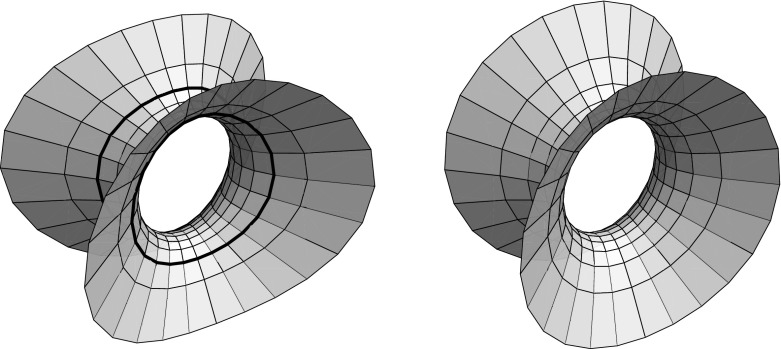
Parametrizations of a 1-sheeted hyperboloid in ${\mathbb R}^3$ resp ${\mathbb R}^{2,1}$ obtained from (5.4)

Note that the same arguments that prove the corresponding properties of the ${\mathbb L}$-Jacobi functions in Def & Cor A.2 also show that this ${\mathbb L}$-analytic extension indeed solves the para-holomorphic differential Eq. (5.3). The Lorentz-analytic function *y* then yields a curvature line parametrization of (part of) the 1-sheeted hyperboloid, cf. Fig. 5.

As the differential Eq. (5.4) that *y* now satisfies only differs by a sign from (4.4) we read the integrals in (5.1) off (4.5),

5.6 $$\begin{aligned} \int {1+y^2\over y'^2}\,dy= & {} {2\over p}\, \arctan {2y\over p(1-y^2)}, \nonumber \\ j\int {1-y^2\over y'^2}\,dy= & {} {2j\over pq}\arctan {2qy\over p(1+y^2)}, \nonumber \\ \int {2y\over y'^2}\,dy= & {} -{2\over q}\,\mathop {\mathrm{artanh}}{q(1-y^2)\over (1+y^2)}. \end{aligned}$$

In case of a complex elliptic modulus, $p\in i{\mathbb R}$, the terms may be turned into purely para-complex form by replacing $\arctan$ by $\mathop {\mathrm{artanh}}$, cf. “Appendix”. Again we obtain two qualitatively different implicit representations for the timelike minimal surfaces $x^*$ given by (5.4), where the $x_1$-axis is now the distinguished axis:

5.7 $$\begin{aligned}&q^2\cos \mathop {\mathrm{Re}}pz_1 +\cos \mathop {\mathrm{Re}}pqz_2 - p^2\cosh \mathop {\mathrm{Re}}qz_3 = 0 \quad \mathrm{if}\;p\in (0,1) \;\mathrm{and}\; q\in (0,1); \nonumber \\&q^2\cosh \mathop {\mathrm{Re}}{pz_1\over i} + \cosh \mathop {\mathrm{Re}}{pqz_2\over i} + p^2\cosh \mathop {\mathrm{Re}}qz_3 = 0 \quad \mathrm{if}\; p\in i{\mathbb R}\;{\mathrm{and}}\;q\in (1,\infty ).\nonumber \\ \end{aligned}$$

Thus, analogous to Cor 4.2, we obtain a “permutability” result for the Christoffel dual of a 1-sheeted hyperboloid in Minkowski space:

### Cor 5.1

The Christoffel dual of (part of) a tri-axial 1-sheeted hyperboloid (5.2) in Minkowski space,

$$\begin{aligned} u+jv=z\mapsto \alpha x(z) = {1\over \mathop {\mathrm{sn}}\nolimits _qu\mathop {\mathrm{dn}}\nolimits _qv} \left( \begin{array}{c} a\mathop {\mathrm{cn}}\nolimits _qu \\ -b\mathop {\mathrm{sn}}\nolimits _qv\mathop {\mathrm{dn}}\nolimits _qu \\ c\mathop {\mathrm{cn}}\nolimits _qv \\ \end{array}\right) \;\mathrm{with}\; q := \sqrt{b^2-c^2\over a^2-c^2}, \end{aligned}$$

is the affine image of a timelike minimal surface, more precisely, up to homothety it is given by

$$\begin{aligned} z\mapsto \alpha x^*(z) := \mathop {\mathrm{Re}}\, \left( \begin{array}{c} -{2a\over p}\arctan {\mathop {\mathrm{cn}}\nolimits _p\over p\mathop {\mathrm{sn}}\nolimits _p}\\ -j\,{2b\over pq}\arctan {q\mathop {\mathrm{cn}}\nolimits _p\over p}\\ {2c\over q}\mathop {\mathrm{artanh}}q\mathop {\mathrm{sn}}\nolimits _p \end{array}\right) (z) \;\mathrm{with}\; p := \sqrt{a^2-b^2\over a^2-c^2}, \end{aligned}$$

where $z=u+jv\in {\mathbb L}$ is a para-complex variable, and $\mathop {\mathrm{cn}}\nolimits _p$ and $\mathop {\mathrm{sn}}\nolimits _p$ denote the ${\mathbb L}$-Jacobi functions obtained as ${\mathbb L}$-analytic extensions of the respective real Jacobi elliptic functions.

To investigate the singularities of the curvature line net, that is, umbilics of the 1-sheeted hyperboloid, we seek again the branch values of the differential Eq. (5.3) resp (5.4):

$$\begin{aligned} 0 = p^2y^4 + 2\left( 1+q^2\right) y^2 + p^2 \;\Leftrightarrow \; y^2 = \left\{ \begin{array}{l} -\left( {1\pm q\over p}\right) ^2 = -\left( {\sqrt{a^2-c^2}\pm \sqrt{b^2-c^2} \over \sqrt{a^2-b^2}}\right) ^2, \\ -\left( {1\pm jq\over p}\right) ^2 = -\left( {\sqrt{a^2-c^2}\pm j\sqrt{b^2-c^2} \over \sqrt{a^2-b^2}}\right) ^2. \end{array}\right. \end{aligned}$$

Thus in order for the branch values to exist (as para-complex numbers) we must have

$$\begin{aligned} {1\pm q\over p} = {\sqrt{a^2-c^2}\pm \sqrt{b^2-c^2}\over \sqrt{a^2-b^2}} \in i{\mathbb R}\;\Leftrightarrow \; \left\{ \begin{array}{ll} c> a> b &{} \mathrm{or} \\ b> a > c. &{} \\ \end{array}\right. \end{aligned}$$

**Fig. 6:**
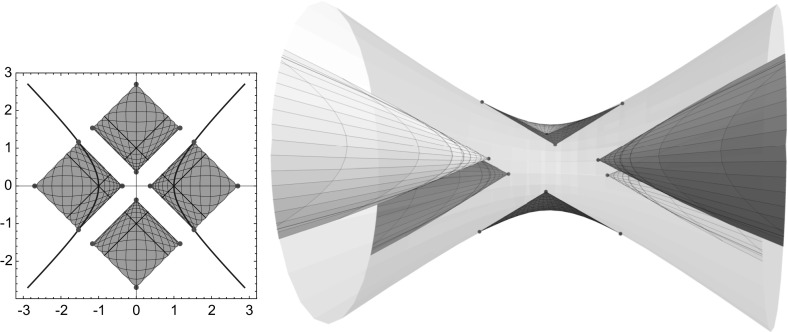
Parametrizations of patches of a 1-sheeted hyperboloid in ${\mathbb R}^{2,1}$ with umbilics

As the para-complex equation $y^2=1$ has four solutions, $y=\pm 1$ and $y=\pm j$, we obtain 16 branch values in this case:

$$\begin{aligned} \pm {q\pm 1\over ip}, \; \pm {q\pm j\over ip}, \; \pm j{q\pm 1\over ip}, \; \pm j{q\pm j\over ip}, \end{aligned}$$

four of which do not project to the hyperboloid since $\Vert {q+j\over ip}\Vert ^2={q+j\over ip}{q-j\over ip}=1$, see Fig. 6. At the same time, the branch values yield the corners of the range of the solution *y* of (5.5), as well as of the solutions ${-}y$ and ${\pm }jy$, obtained from *y* by symmetry.

This sharply contrasts the Euclidean case, where the orthogonality condition leads to the differential equation

5.8 $$\begin{aligned} y'^2 = \varrho \,\left\{ a^2\left( 1+y^2\right) ^2 + b^2\left( 1-y^2\right) ^2 + 4c^2y^2 \right\} , \end{aligned}$$

cf. (2.3) and (4.7). As a para-complex differential equations this does not admit any branch values—not surprisingly, as a 1-sheeted hyperboloid in Euclidean space cannot have any umbilics.

Following our earlier strategy we obtain an explicit solution by replacing *a* by *ia* in (5.4); the corresponding curvature line parametrization of the hyperboloid and its dual is then simply read off Cor 5.1:

### Cor 5.2

A tri-axial 1-sheeted hyperboloid $1+({x_1\over a})^2=({x_2\over b})^2+({x_3\over c})^2$ in Euclidean space, where without loss of generality $b<c$, and its Christoffel dual admit curvature line parametrizations by ${\mathbb L}$-analytic extensions of Jacobi elliptic functions:

$$\begin{aligned} \alpha x(z) = {1\over \mathop {\mathrm{sn}}\nolimits _qu\mathop {\mathrm{dn}}\nolimits _qv} \left( \begin{array}{c} a\mathop {\mathrm{cn}}\nolimits _qu \\ -b\mathop {\mathrm{sn}}\nolimits _qv\mathop {\mathrm{dn}}\nolimits _qu \\ c\mathop {\mathrm{cn}}\nolimits _qv \\ \end{array}\right) \;\mathrm{and}\; \alpha x^*(z) = \mathop {\mathrm{Re}}\, \left( \begin{array}{c} -{2a\over p}\arctan {\mathop {\mathrm{cn}}\nolimits _p\over p\mathop {\mathrm{sn}}\nolimits _p}\\ -j\,{2b\over pq}\arctan {q\mathop {\mathrm{cn}}\nolimits _p\over p}\\ {2c\over q}\mathop {\mathrm{artanh}}q\mathop {\mathrm{sn}}\nolimits _p \end{array}\right) (z). \end{aligned}$$

where $z=u+jv\in {\mathbb L}$, and $p=\sqrt{a^2+b^2\over a^2+c^2}$ and $q=\sqrt{-{b^2-c^2\over a^2+c^2}}$, cf. Fig. 5.

## Appendix: Reinbek’s formulas

It has been known for long that quadrics are isothermic surfaces, in fact, it seems that investigations on quadrics were one of the main motivations to investigate isothermic surfaces, cf. [1]. After obtaining the Christoffel duality for isothermic surfaces in [2] an obvious question was to determine the Christoffel transforms of quadrics: this was the topic of the Ph.D. thesis [15].

As this thesis may not be very easy to obtain or read we briefly list the results that are most relevant to this paper: using the well known conversion formulas

$$\begin{aligned} \mathop {\mathrm{artanh}}t = {1\over 2}\ln {1+t\over 1-t} \;\mathrm{and}\; \arctan t = {1\over 2i}\ln {1+it\over 1-it} \end{aligned}$$

we rewrite the representations that Reinbek obtained for the Christoffel duals of quadrics in terms of the usual elliptic coordinates in [15, §§6,7,8]:

(i) for an ellipsoid $\{ (x_1,x_2,x_3)\,|\, ({x_1\over a})^2+({y\over b})^2+({z\over c})^2=1\}$, with $a^2\ge t_1\ge b^2\ge t_2\ge c^2$,
  $$\begin{aligned} x(t_1,t_2)= & {} \left( \begin{array}{c} a\,\sqrt{\left( a^2-t_1\right) \left( a^2-t_2\right) \over \left( a^2-b^2\right) \left( a^2-c^2\right) } \\ b\,\sqrt{\left( b^2-t_1\right) \left( b^2-t_2\right) \over \left( b^2-a^2\right) \left( b^2-c^2\right) } \\ c\,\sqrt{\left( c^2-t_1\right) \left( c^2-t_2\right) \over \left( c^2-a^2\right) \left( c^2-b^2\right) } \end{array}\right) \;\mathrm{and}\\ x^*(t_1,t_2)= & {} \left( \begin{array}{c} \sqrt{a^2\over \left( a^2-b^2\right) \left( a^2-c^2\right) } \mathop {\mathrm{artanh}}\sqrt{a^2-t_1\over a^2-t_2} \\ \sqrt{b^2\over \left( a^2-b^2\right) \left( b^2-c^2\right) } \arctan \sqrt{t_1-b\over b^2-t_2} \\ \sqrt{c^2\over \left( a^2-c^2\right) \left( b^2-c^2\right) } \mathop {\mathrm{artanh}}\sqrt{t_2-c^2\over t_1-c^2} \end{array}\right) ; \end{aligned}$$
(ii) for a hyperboloid $\{ (x_1,x_2,x_3)\,|\, ({x_1\over a})^2+({y\over b})^2-({z\over c})^2=1\}$, with $a^2\ge t_1\ge b^2 > -c^2\ge t_2$,
  $$\begin{aligned} x(t_1,t_2)= & {} \left( \begin{array}{c} a\,\sqrt{\left( a^2-t_1\right) \left( a^2-t_2\right) \over \left( a^2-b^2\right) \left( a^2+c^2\right) } \\ b\,\sqrt{\left( b^2-t_1\right) \left( b^2-t_2\right) \over \left( b^2-a^2\right) \left( b^2+c^2\right) } \\ c\,\sqrt{\left( c^2+t_1\right) \left( c^2+t_2\right) \over \left( c^2+a^2\right) \left( c^2+b^2\right) } \end{array}\right) \;\mathrm{and}\\ x^*(t_1,t_2)= & {} \left( \begin{array}{c} \sqrt{a^2\over \left( a^2-b^2\right) \left( a^2+c^2\right) } \mathop {\mathrm{artanh}}\sqrt{a^2-t_1\over a^2-t_2} \\ \sqrt{b^2\over \left( a^2-b^2\right) \left( b^2+c^2\right) } \arctan \sqrt{t_1-b^2\over b^2-t_2} \\ \sqrt{c^2\over \left( a^2+c^2\right) \left( b^2+c^2\right) } \arctan \sqrt{-{t_1+c^2\over c^2+t_2}} \end{array}\right) ; \end{aligned}$$
(iii) for a hyperboloid $\{ (x_1,x_2,x_3)\,|\, ({x_1\over a})^2-({y\over b})^2-({z\over c})^2=1\}$, with $a^2>-b^2\ge t_1\ge -c^2\ge t_2$,
  $$\begin{aligned} x(t_1,t_2)= & {} \left( \begin{array}{c} a\,\sqrt{\left( a^2-t_1\right) \left( a^2-t_2\right) \over \left( a^2+b^2\right) \left( a^2+c^2\right) } \\ b\,\sqrt{\left( b^2+t_1\right) \left( b^2+t_2\right) \over \left( b^2+a^2\right) \left( b^2-c^2\right) } \\ c\,\sqrt{\left( c^2+t_1\right) \left( c^2+t_2\right) \over \left( c^2+a^2\right) \left( c^2-b^2\right) } \end{array}\right) \;\mathrm{and}\\ x^*(t_1,t_2)= & {} \left( \begin{array}{c} \sqrt{a^2\over \left( a^2+b^2\right) \left( a^2+c^2\right) } \mathop {\mathrm{artanh}}\sqrt{a^2-t_1\over a^2-t_2} \\ \sqrt{b^2\over \left( a^2+b^2\right) \left( -b^2+c^2\right) } \mathop {\mathrm{artanh}}\sqrt{b^2+t_1\over b^2+t_2} \\ \sqrt{c^2\over \left( a^2+c^2\right) \left( -b^2+c^2\right) } \arctan \sqrt{-{t_1+c^2\over c^2+t_2}} \end{array}\right) . \end{aligned}$$

## Lorentz analytic functions

Our analysis of the Christoffel duals of quadrics and the associated zero mean curvature surfaces hinges crucially on elliptic functions, as solutions of the occurring elliptic differential equations. To obtain the para-holomorphic analogues of the Jacobi elliptic functions we seek a suitable extension theorem for para-holomorphic functions.

Following [4, Def 4.11(4)] we say that a function $y:{\mathbb L}\supset U\rightarrow {\mathbb L}$ is ${\mathbb L}$ *-analytic* (or *Lorentz-analytic*) if it admits a power series expansion around every point $z_0=u_0+jv_0\in U$,

$$\begin{aligned} y(u+jv) = \sum \limits _{n\in {\mathbb N}}a_n\left( (u-u_0)+j(v-v_0)\right) ^n. \end{aligned}$$

Any ${\mathbb L}$-analytic function is para-holomorphic in its domain, in fact, has para-complex derivatives of any order, which are given by term-by-term differentiation of its power series [4, Thm 4.12].

Note that, in contrast to the case of complex holomorphic functions, para-holomorphicity does neither imply ${\mathbb L}$-analyticity nor the existence of higher order derivatives, cf. [4, Expl 4.13].

In fact, any differentiable function $y:{\mathbb R}\supset I\rightarrow {\mathbb R}$, regardless of higher order differentiability of *y*, has a para-holomorphic extension

$$\begin{aligned} \tilde{y}(u+jv) : = y(u+v)\,{1+j\over 2} + y(u-v)\,{1-j\over 2} \end{aligned}$$

as $\mathop {\mathrm{Re}}\tilde{y}(u,v) = {1\over 2}(y(u+v)+y(u-v))$ and $\mathop {\mathrm{Im}}\tilde{y}(u,v) = {1\over 2}(y(u+v)-y(u-v))$ clearly satisfy the para-complex version of the Cauchy–Riemann equations,

$$\begin{aligned} (\mathop {\mathrm{Re}}\tilde{y})_u = (\mathop {\mathrm{Im}}\tilde{y})_v \;\mathrm{and}\; (\mathop {\mathrm{Im}}\tilde{y})_u = (\mathop {\mathrm{Re}}\tilde{y})_v. \end{aligned}$$

Note that this extension is not unique: for example, $j\tilde{y}$ is para-holomorphic with $j\tilde{y}|_I\equiv 0$, hence any superposition of $\tilde{y}$ and $j\tilde{y}$ yields another extension of *y*; in fact, any real differentiable function *y* provides a para-holomorphic extension $j\tilde{y}$ of 0.

To see that the above construction yields a unique extension of a real analytic function to an ${\mathbb L}$-analytic function, first observe that

$$\begin{aligned} \left( {1\pm j\over 2}\right) ^2 = {1\pm j\over 2} \;\mathrm{and}\; \left( {1+j\over 2}\right) \left( {1-j\over 2}\right) = 0. \end{aligned}$$

Thus writing $u+jv=(u+v){1+j\over 2}+(u-v){1-j\over v}$ we learn that the extension of a real power series is a para-complex power series since

$$\begin{aligned} \sum \limits _{n\in {\mathbb N}}a_n(u+jv)^n = \sum \limits _{n\in {\mathbb N}}a_n(u+v)^n{1+j\over 2} + \sum \limits _{n\in {\mathbb N}}a_n(u-v)^n{1-j\over 2}. \end{aligned}$$

Uniqueness follows by comparison of coefficients: if $\tilde{y}(u+jv)=\sum _{n\in {\mathbb N}}a_n(u+jv)^n$ restricts to a real analytic function *y* then all coefficients $a_n$ must be real, hence are uniquely determined by *y*.

Thus we have proved the following, cf. [4, Expl 3.2 and Prop 3.1(4)]:

### Lemma A.1

Every real analytic function $y:{\mathbb R}\supset I\rightarrow {\mathbb R}$ has a unique ${\mathbb L}$-analytic extension

$$\begin{aligned}&\tilde{y}:{\mathbb L}\supset \left\{ u+jv\,|\,u\pm v\in I\right\} \rightarrow {\mathbb L}, \;\\&\quad u+jv\mapsto \tilde{y}(u+jv) := y(u+v)\,{1+j\over 2} + y(u-v)\,{1-j\over 2}. \end{aligned}$$

Note that the ${\mathbb L}$-analytic trigonometric functions of [4, Expl 3.3] are different from those that are obtained by extension of the real trigonometric functions.

Clearly, sums and products of the ${\mathbb L}$-analytic extensions of real analytic functions are the ${\mathbb L}$-analytic extensions of the corresponding sums resp products of the real functions, and as

$$\begin{aligned} \left( y(u+v){1+j\over 2}+y(u-v){1-j\over 2}\right) \left( {1\over y(u+v)}{1+j\over 2}+{1\over y(u-v)}{1-j\over 2}\right) = 1 \end{aligned}$$

the same holds true for quotients, where they are defined. In fact, these properties also follow from the compatibility of our ${\mathbb L}$-analytic extension with the composition,

$$\begin{aligned} \widetilde{f\circ y} = \tilde{f}\circ \tilde{y} \;\mathrm{since}\; \left( \mathop {\mathrm{Re}}\tilde{y}\pm \mathop {\mathrm{Im}}\tilde{y}\right) (u+jv) = y(u\pm v). \end{aligned}$$

the composition of the extensions of real analytic functions

As the derivative of an ${\mathbb L}$-analytic function is obtained by term-by-term differentiation of its power series expansion it is clear that the derivative of the ${\mathbb L}$-analytic extension of a real analytic function is the ${\mathbb L}$-analytic extension of its derivative. However, a similar argument as above can be given, that does not depend on the power series expansion:

$$\begin{aligned} \tilde{y}'(u+jv)= & {} {1\over 2} \left( {\partial \over \partial u}+j{\partial \over \partial v}\right) \, \tilde{y}(u+jv) \\= & {} {y'+jy'\over 2}(u+v){1+j\over 2} + {y'-jy'\over 2}(u+v){1-j\over 2} = \widetilde{y'}(u+jv). \end{aligned}$$

## Jacobi elliptic functions

For the reader’s convenience we collect some basic facts about the Jacobi elliptic functions of pole type *n*: in terms of the Jacobi amplitude function $\mathop {\mathrm{am}}\nolimits _p$ these may be defined by

$$\begin{aligned} \mathop {\mathrm{cn}}\nolimits _pz = \cos \mathop {\mathrm{am}}\nolimits _pz, \; \mathop {\mathrm{sn}}\nolimits _pz = \sin \mathop {\mathrm{am}}\nolimits _pz, \; \mathop {\mathrm{dn}}\nolimits _pz = \sqrt{1-p^2\mathop {\mathrm{sn}}\nolimits _p^2z}. \end{aligned}$$

The Pythagorean laws are then a direct consequence of these definitions:

$$\begin{aligned} 1 = \mathop {\mathrm{cn}}\nolimits _p^2 + \mathop {\mathrm{sn}}\nolimits _p^2 = \mathop {\mathrm{dn}}\nolimits _p^2 + p^2\mathop {\mathrm{sn}}\nolimits _p^2; \end{aligned}$$

and with $\mathop {\mathrm{am}}\nolimits _p'=\mathop {\mathrm{dn}}\nolimits _p$ the characterizing elliptic differential equations are readily derived:

$$\begin{aligned} \mathop {\mathrm{cn}}\nolimits _p'^2= & {} \left( 1-\mathop {\mathrm{cn}}\nolimits _p^2\right) \left( q^2+p^2\mathop {\mathrm{cn}}\nolimits _p^2\right) ,\;\\ \mathop {\mathrm{sn}}\nolimits _p'^2= & {} \left( 1-\mathop {\mathrm{sn}}\nolimits _p^2\right) \left( 1-p^2\mathop {\mathrm{sn}}\nolimits _p^2\right) , \;\\ \mathop {\mathrm{dn}}\nolimits _p'^2= & {} \left( \mathop {\mathrm{dn}}\nolimits _p^2-1\right) \left( q^2-\mathop {\mathrm{dn}}\nolimits _p^2\right) . \end{aligned}$$

The Jacobi elliptic functions are analytic functions, naturally defined for complex arguments: in fact, they are holomorphic functions from a torus to the Riemann sphere since they are doubly periodic, with the period lattice spanned by $4K_p$ and $4iK_q$, where $K_p$ denotes the complete elliptic integral of the first kind.

To resort to real arguments the conversion formulae of imaginary arguments,

$$\begin{aligned} \mathop {\mathrm{cn}}\nolimits _p(iz) = {1\over \mathop {\mathrm{cn}}\nolimits _qz}, \; \mathop {\mathrm{sn}}\nolimits _p(iz) = i\,{\mathop {\mathrm{sn}}\nolimits _qz\over \mathop {\mathrm{cn}}\nolimits _qz}, \; \mathop {\mathrm{dn}}\nolimits _p(iz) = {\mathop {\mathrm{dn}}\nolimits _qz\over \mathop {\mathrm{cn}}\nolimits _qz}, \end{aligned}$$

are combined with the argument sum formulas

$$\begin{aligned} \mathop {\mathrm{cn}}\nolimits _p(u+v)= & {} {\mathop {\mathrm{cn}}\nolimits _pu\mathop {\mathrm{cn}}\nolimits _pv-\mathop {\mathrm{sn}}\nolimits _pu\mathop {\mathrm{dn}}\nolimits _pu\mathop {\mathrm{sn}}\nolimits _pv\mathop {\mathrm{dn}}\nolimits _pv \over 1-p^2\mathop {\mathrm{sn}}\nolimits _p^2u\mathop {\mathrm{sn}}\nolimits _p^2v}, \\ \mathop {\mathrm{sn}}\nolimits _p(u+v)= & {} { \mathop {\mathrm{sn}}\nolimits _pu\mathop {\mathrm{cn}}\nolimits _pv\mathop {\mathrm{dn}}\nolimits _pv+\mathop {\mathrm{sn}}\nolimits _pv\mathop {\mathrm{cn}}\nolimits _pu\mathop {\mathrm{dn}}\nolimits _pu \over 1-p^2\mathop {\mathrm{sn}}\nolimits _p^2u\mathop {\mathrm{sn}}\nolimits _p^2v}, \\ \mathop {\mathrm{dn}}\nolimits _p(u+v)= & {} { \mathop {\mathrm{dn}}\nolimits _pu\mathop {\mathrm{dn}}\nolimits _pv-p^2\mathop {\mathrm{sn}}\nolimits _pu\mathop {\mathrm{cn}}\nolimits _pu\mathop {\mathrm{sn}}\nolimits _pv\mathop {\mathrm{cn}}\nolimits _pv \over 1-p^2\mathop {\mathrm{sn}}\nolimits _p^2u\mathop {\mathrm{sn}}\nolimits _p^2v}. \\ \end{aligned}$$

Hence, for example,

$$\begin{aligned} e^{i\mathop {\mathrm{am}}\nolimits _p(u+iv)} = {\mathop {\mathrm{cn}}\nolimits _pu\mathop {\mathrm{cn}}\nolimits _qv+i\mathop {\mathrm{sn}}\nolimits _pu\mathop {\mathrm{dn}}\nolimits _qv\over 1+\mathop {\mathrm{dn}}\nolimits _pu\mathop {\mathrm{sn}}\nolimits _qv}. \end{aligned}$$

Finally, the conversion formulas for inverse and imaginary moduli allow to unify the treatment of hyperboloids of different half axes configurations:

$$\begin{aligned} \begin{array}{ll} \mathop {\mathrm{cn}}\nolimits _{1/p}z = \mathop {\mathrm{dn}}\nolimits _p{z\over p}, &{}\; \mathop {\mathrm{cn}}\nolimits _{ip}z = {\mathop {\mathrm{cn}}\nolimits _{p'}\over \mathop {\mathrm{dn}}\nolimits _{p'}}{z\over q'}, \\ \mathop {\mathrm{sn}}\nolimits _{1/p}z = p\mathop {\mathrm{sn}}\nolimits _p{z\over p}, &{}\; \mathop {\mathrm{sn}}\nolimits _{ip}z = q'{\mathop {\mathrm{sn}}\nolimits _{p'}\over \mathop {\mathrm{dn}}\nolimits _{p'}}{z\over q'}, \\ \mathop {\mathrm{dn}}\nolimits _{1/p}z = \mathop {\mathrm{cn}}\nolimits _p{z\over p}; &{}\; \mathop {\mathrm{dn}}\nolimits _{ip}z = {1\over \mathop {\mathrm{dn}}\nolimits _{p'}}{z\over q'}, \end{array} \;\mathrm{where}\; \left\{ \begin{array}{l} p'={p\over \sqrt{1+p^2}}, \\ q'={1\over \sqrt{1+p^2}}. \end{array}\right. \end{aligned}$$

Note that these conversions preserve reality of the Jacobi elliptic functions for real arguments.

Considering the Jacobi elliptic functions as real analytic functions we may use Lemma A.1 to obtain ${\mathbb L}$-analytic extensions that are defined on a square torus:

### Def & Cor A.2

The Jacobi elliptic functions have unique ${\mathbb L}$-analytic extensions to ${\mathbb L}$, that we will refer to as ${\mathbb L}$ *-elliptic* or ${\mathbb L}$ *-Jacobi functions*. These ${\mathbb L}$-elliptic functions satisfy the same elliptic differential equations as their real counterparts and they are doubly periodic, with fundamental domains

$$\begin{aligned} \left\{ u+jv\,|\, -2K_p\le u\pm v\le 2K_p \right\} . \end{aligned}$$

Existence of unique ${\mathbb L}$-analytic extensions and their periodicity follows directly from Lemma A.1; as the above elliptic differential equations characterizing the Jacobi elliptic functions are (real) analytic,

$$\begin{aligned} 0 = f(y',y) \;\mathrm{with}\; f\in C^\omega ({\mathbb R}^2), \end{aligned}$$

the extensions of $y'$ and *y* satisfy the same elliptic differential equations as the original real functions do. For example, the real solution

$$\begin{aligned} y(u) = e^{i\mathop {\mathrm{am}}\nolimits _piu} = {\mathop {\mathrm{cn}}\nolimits _qu\over 1+\mathop {\mathrm{sn}}\nolimits _qu} \;\mathrm{of}\; y'^2 = {1\over 4}\left\{ p^2(1+y^4)+2(1+q^2)\,y^2 \right\} \end{aligned}$$

extends to a para-complex solution of the same elliptic differential equation,

$$\begin{aligned} y(u+jv) = {\mathop {\mathrm{cn}}\nolimits _qu-j\mathop {\mathrm{sn}}\nolimits _qv\mathop {\mathrm{dn}}\nolimits _qu \over \mathop {\mathrm{cn}}\nolimits _qv+\mathop {\mathrm{sn}}\nolimits _qu\mathop {\mathrm{dn}}\nolimits _qv}. \end{aligned}$$

Note that $-y$ and $\pm jy$ provide other solutions of the same elliptic differential equation.

## Implicit representations

While verification of the implicit representations of the occurring zero mean curvature surfaces given in the text is fairly straightforward, we provide some hints and formulas that may facilitate the endeavour in this appendix. First recall that three cases are discussed:

**Table:**

Case	Elliptic ode	Solution
Ellipsoid/Scherk towers (3.3)	$y'^2 = {(1-y^2)^2 - q^2(1+y^2)^2\over 4}$	$y(z)={1\over i}\,e^{i\mathop {\mathrm{am}}\nolimits _pz}$,
2-sheeted hyperboloid (4.4)	$-y'^2 = {(1+y^2)^2 - q^2(1-y^2)^2\over 4}$	$y(z)=e^{i\mathop {\mathrm{am}}\nolimits _pz}$,
1-sheeted hyperboloid (5.4)	$y'^2= {(1+y^2)^2 - q^2(1-y^2)^2\over 4}$	$y(z) = e^{i\mathop {\mathrm{am}}\nolimits _piz}$.

Here $p=\sqrt{{a^2-b^2\over a^2-c^2}}$ and $q=\sqrt{1-p^2}=\sqrt{{b^2-c^2\over a^2-c^2}}$ denote the elliptic modulus and co-modulus of the elliptic function *y*, obtained from the half axes $a,b,c>0$ of the ellipsoid or hyperboloid, respectively. Note that, depending on the order of the half axes, three cases can occur:

$$\begin{aligned} \begin{array}{llllllll} \mathrm{(i)} &{} a>b>c &{}\mathrm{or}&{} c>b>a &{}\mathrm{yields}&{} p\in (0,1) &{}\mathrm{and}&{} q\in (0,1); \\ \mathrm{(ii)} &{} c>a>b &{}\mathrm{or}&{} b>a>c &{}\mathrm{yields}&{} p\in i{\mathbb R}&{}\mathrm{and}&{} q>1; \\ \mathrm{(iii)} &{} b>c>a &{}\mathrm{or}&{} a>c>b &{}\mathrm{yields}&{} p>1 &{}\mathrm{and}&{} q\in i{\mathbb R}. \\ \end{array} \end{aligned}$$

Using the differential equation of the elliptic function *y* the integrals in the Weierstrass representations, (3.4), (4.5) and (5.6), are readily verified using

$$\begin{aligned} \begin{array}{llllll} \left( \mathop {\mathrm{artanh}}{2y\over p\left( 1+y^2\right) }\right) ' &{}=&{} {2y'p\left( 1-y^2\right) \over \left( 1-y^2\right) ^2-q^2\left( 1+y^2\right) ^2}, &{}\;\; \left( \arctan {2y\over p(1-y^2)}\right) ' &{}=&{} {2y'p\left( 1+y^2\right) \over \left( 1+y^2\right) ^2-q^2\left( 1-y^2\right) ^2}; \\ \left( \mathop {\mathrm{artanh}}{2qy\over p\left( 1-y^2\right) }\right) ' &{}=&{} {2y'pq\left( 1+y^2\right) \over \left( 1-y^2\right) ^2-q^2\left( 1+y^2\right) ^2}, &{}\;\; \left( \arctan {2qy\over p\left( 1+y^2\right) }\right) ' &{}=&{} {2y'pq\left( 1-y^2\right) \over \left( 1+y^2\right) ^2-q^2\left( 1-y^2\right) ^2}; \\ \left( \mathop {\mathrm{artanh}}{q\left( 1\pm y^2\right) \over \left( 1\mp y^2\right) }\right) ' &{}=&{} {\pm 4yy'q\,\over \left( 1\mp y^2\right) ^2-q^2\left( 1\pm y^2\right) ^2}. \\ \end{array} \end{aligned}$$

We now investigate the three cases, the ellipsoid in Euclidean space and the space- resp timelike hyperboloids in Minkowski geometry, in turn.

Ellipsoid/Scherk tower (3.4): We set

$$\begin{aligned} z_1:= & {} \int {1-y^2\over y'^2}\,dy ={2\over p}\,\mathop {\mathrm{artanh}}{2y\over p(1+y^2)}, \nonumber \\ z_2:= & {} i\int {1+y^2\over y'^2}\,dy = {2i\over pq}\mathop {\mathrm{artanh}}{2qy\over p(1-y^2)} = {2\over pq}\,\arctan {2iqy\over p(1-y^2)}, \nonumber \\ z_3:= & {} \int {2y\over y'^2}\,dy ={2\over q}\,\mathop {\mathrm{artanh}}{q(1+y^2)\over (1-y^2)}. \end{aligned}$$

Then we use

$$\begin{aligned} \cosh (z+\bar{z}) = {1+|\tanh z|^2\over |1-\tanh ^2z|} \;\mathrm{and}\; \cos (z+\bar{z}) = {1-|\tan z|^2\over |1+\tan ^2 z|} \end{aligned}$$

to compute

$$\begin{aligned} \begin{array}{lllllll} \tanh ^2{pz_1\over 2} = {4y^2\over p^2\left( 1+y^2\right) ^2}, &{} \cosh \mathop {\mathrm{Re}}pz_1 = {p^2|1+y^2|^2+4|y|^2 \over \left| \left( 1-y^2\right) ^2-q^2\left( 1+y^2\right) ^2\right| }; \\ \tan ^2{pqz_2\over 2} = {-4q^2y^2\over p^2\left( 1-y^2\right) ^2}, &{} \cos \mathop {\mathrm{Re}}pqz_2 = {p^2|1-y^2|^2-4q^2|y|^2 \over \left| \left( 1-y^2\right) ^2-q^2\left( 1+y^2\right) ^2\right| }; \\ \tanh ^2{qz_3\over 2} = {q^2\left( 1+y^2\right) ^2\over \left( 1-y^2\right) ^2}, &{} \cosh \mathop {\mathrm{Re}}qz_3 = {|1-y^2|^2+q^2|1+y^2|^2 \over \left| \left( 1-y^2\right) ^2-q^2\left( 1+y^2\right) ^2\right| }. \\ \end{array} \end{aligned}$$

Without loss of generality we assume case (i) $a>b>c$ here, so that $p,q\in (0,1)$. Thus we obtain the implicit representation (3.5) of the Scherk tower $x^*$ of Lemma 3.1:

$$\begin{aligned} q^2\cosh \mathop {\mathrm{Re}}pz_1 + \cos \mathop {\mathrm{Re}}pqz_2 - p^2\cosh \mathop {\mathrm{Re}}qz_3 = 0. \end{aligned}$$

2-sheeted hyperboloid (4.5): Here we set

$$\begin{aligned} z_1:= & {} \int {1+y^2\over y'^2}\,dy =-{2\over p}\,\arctan {2y\over p(1-y^2)}, \nonumber \\ z_2:= & {} i\int {1-y^2\over y'^2}\,dy = -{2i\over pq}\arctan {2qy\over p(1+y^2)} = {2\over pq}\,\mathop {\mathrm{artanh}}{2qy\over ip(1+y^2)}, \nonumber \\ z_3:= & {} \int {2y\over y'^2}\,dy ={2\over q}\,\mathop {\mathrm{artanh}}{q(1-y^2)\over (1+y^2)} ={2i\over q}\,\arctan {q(1-y^2)\over i(1+y^2)}. \end{aligned}$$

Employing the same method as before, two cases, (i) $a>b>c$ and (iii) $b>c>a$, need to be considered, as the $x_3$-axis is geometrically distinguished: exchanging the $x_1$- and $x_2$-axes will interchange the equations of cases (i) and (ii). Thus we compute

and hence obtain implicit representations (4.6) for the maximal surfaces of Cor 4.2:

$$\begin{aligned}&q^2\cos \mathop {\mathrm{Re}}pz_1 + \cosh \mathop {\mathrm{Re}}pqz_2 - p^2\cosh \mathop {\mathrm{Re}}qz_3 = 0 \quad \mathrm{if}\; p<1 \;\mathrm{and}\; q\in (0,1); \\&q^2\cos \mathop {\mathrm{Re}}pz_1 + \cos \mathop {\mathrm{Re}}{pqz_2\over i} - p^2\cos \mathop {\mathrm{Re}}{qz_3\over i} = 0 \quad \mathrm{if}\; p>1 \;\mathrm{and}\; q\in i{\mathbb R}. \end{aligned}$$

1-sheeted hyperboloid (5.6): Here we use para-complex coordinate functions

$$\begin{aligned} z_1:= & {} \int {1+y^2\over y'^2}\,dy ={2\over p}\,\arctan {2y\over p(1-y^2)} =-{2i\over p}\,\mathop {\mathrm{artanh}}{2iy\over p(1-y^2)}, \\ z_2:= & {} j\int {1-y^2\over y'^2}\,dy = {2j\over pq}\arctan {2qy\over p(1+y^2)} = -{2ij\over pq}\mathop {\mathrm{artanh}}{2iqy\over p(1+y^2)}, \\ z_3:= & {} \int {2y\over y'^2}\,dy = -{2\over q}\,\mathop {\mathrm{artanh}}{q(1-y^2)\over (1+y^2)}. \end{aligned}$$

Note that we are now using the ${\mathbb L}$-analytic extensions of real elliptic functions, hence may need to replace $\mathop {\mathrm{artanh}}$ by $\mathrm{arcoth}$ if necessary—however, this has no effect as we again use

$$\begin{aligned} \cosh (z+\bar{z}) = {1+\Vert \tanh z\Vert ^2\over \Vert 1-\tanh ^2z\Vert } \;\mathrm{and}\; \cos (z+\bar{z}) = {1-\Vert \tan z\Vert ^2\over \Vert 1+\tan ^2 z\Vert }, \end{aligned}$$

together with $\tan jz=j\tan z$, $\tanh jz=j\tanh z$ and $\Vert jy\Vert ^2=-\Vert y\Vert ^2$, to compute

Thus we obtain implicit representations (5.7) of the timelike minimal surfaces of Cor 5.1:

$$\begin{aligned}&q^2\cos \mathop {\mathrm{Re}}pz_1 + \cos \mathop {\mathrm{Re}}pqz_2 - p^2\cosh \mathop {\mathrm{Re}}qz_3 = 0 \quad \mathrm{if}\; p\in (0,1) \;\mathrm{and}\; q\in (0,1); \\&q^2\cosh \mathop {\mathrm{Re}}{pz_1\over i} + \cosh \mathop {\mathrm{Re}}{pqz_2\over i} + p^2\cosh \mathop {\mathrm{Re}}qz_3 = 0 \quad \mathrm{if}\; p\in i{\mathbb R}\;\mathrm{and}\; q\in (1,\infty ). \end{aligned}$$

Note that the third case, (iii) $b>c>a$, can be neglected since the $x_1$-axis is the timelike axis so that this case can be obtained from case (i) by exchanging the $x_2$- and $x_3$-axes.
